# Radiation Dosimetry by Use of Radiosensitive Hydrogels and Polymers: Mechanisms, State-of-the-Art and Perspective from 3D to 4D

**DOI:** 10.3390/gels8090599

**Published:** 2022-09-19

**Authors:** Yves De Deene

**Affiliations:** 1 Liverpool & Macarthur Cancer Therapy Centres, Liverpool, NSW 1871, Australia; yves.dedeene@health.nsw.gov.au or y.dedeene@westernsydney.edu.au; 2 Ingham Institute, Liverpool, NSW 2170, Australia; 3 School of Science, Western Sydney University, Penrith, NSW 2751, Australia

**Keywords:** radiation dosimetry, gel dosimetry, hydrogels, Fricke gel dosimetry, radiochromic dosimetry, polymer gel dosimetry, MR-Linac

## Abstract

Gel dosimetry was developed in the 1990s in response to a growing need for methods to validate the radiation dose distribution delivered to cancer patients receiving high-precision radiotherapy. Three different classes of gel dosimeters were developed and extensively studied. The first class of gel dosimeters is the Fricke gel dosimeters, which consist of a hydrogel with dissolved ferrous ions that oxidize upon exposure to ionizing radiation. The oxidation results in a change in the nuclear magnetic resonance (NMR) relaxation, which makes it possible to read out Fricke gel dosimeters by use of quantitative magnetic resonance imaging (MRI). The radiation-induced oxidation in Fricke gel dosimeters can also be visualized by adding an indicator such as xylenol orange. The second class of gel dosimeters is the radiochromic gel dosimeters, which also exhibit a color change upon irradiation but do not use a metal ion. These radiochromic gel dosimeters do not demonstrate a significant radiation-induced change in NMR properties. The third class is the polymer gel dosimeters, which contain vinyl monomers that polymerize upon irradiation. Polymer gel dosimeters are predominantly read out by quantitative MRI or X-ray CT. The accuracy of the dosimeters depends on both the physico-chemical properties of the gel dosimeters and on the readout technique. Many different gel formulations have been proposed and discussed in the scientific literature in the last three decades, and scanning methods have been optimized to achieve an acceptable accuracy for clinical dosimetry. More recently, with the introduction of the MR-Linac, which combines an MRI-scanner and a clinical linear accelerator in one, it was shown possible to acquire dose maps during radiation, but new challenges arise.

## 1. Introduction

The technological evolution in modern radiotherapy has been driven by the paradigm that delivering radiation to a target volume with high precision while sparing the surrounding healthy tissues leads to an increase in local tumor control while complications are minimized [1]. There exists an increasing body of clinical evidence that the implementation of modern three-dimensional (3D) treatment modalities, such as intensity-modulated therapy (IMRT), intensity modulated arc therapy (IMAT) and tomotherapy, results in a significantly higher cancer treatment efficiency [2]. The increased precision in delivering radiation to a specific target volume also results in a higher demand for treatment accuracy. Indeed, the steep radiation dose gradients around the tumor, in combination with an escalation of dose in the target volume, mean that any geometrical inaccuracy in the delivered radiation beams may result in large radiation doses in healthy tissue, while some parts of the tumor may be underdosed. In routine clinical practice, medical radiation physicists employ a variety of radiation dosimeters to guarantee the accuracy of the radiation delivery. Most radiation dosimeters are only capable of registering the radiation dose in one spatial dimension or two dimensions [3]. Moreover, most of these quality assurance procedures are conducted in canonical radiation conditions such as box-shaped water phantoms. Gel dosimeters are capable of measuring the dose in humanoid shaped phantoms in three dimensions. Gel dosimeters were never intended to replace other radiation dosimeters for treatment machine calibration, but because of their three-dimensional character, they have a unique role in end-to-end verification of modern radiotherapy, especially where dose registration with other dosimeters is problematic because of steep dose gradients in all three dimensions [2]. An ideal 3D radiation dosimeter satisfies the following physical properties:

- *A high dose resolution*: The dose resolution is defined as the minimum dose difference that will be detected by the dosimeter with high certainty (e.g., 95%). The dose resolution depends on both the dose sensitivity of the dosimeter and the readout technique (i.e., the signal-to-noise (SNR) ratio).
- *A high temporal stability* and *a high spatial integrity*. This means that the dose reading should be stable over time and the acquired dose distribution should not change over time. Sources of instability are related to the reaction kinetics, which ideally should be fast. The spatial integrity in chemical dosimeters may be compromised because of the diffusion of chemicals in the gel during and/or after radiation.
- As the dosimeter acts as a surrogate for the human body, the dosimeter should be *tissue equivalent*. The attenuation of the radiation beam by the dosimeter should be similar to human tissue. For high energetic photon radiation, this is mostly satisfied if the electron density of the dosimeter is close to that of the tissue. The tissue equivalence should be guaranteed over a large range of photon energies to cover the photon energy spectrum of linear accelerators (linacs) and other kinds of radiation, such as brachytherapy and orthovoltage treatments.
- *Temperature independent dose response*: The dose response of the dosimeter should not depend on the temperature during radiation, after radiation or during scanning. *Dose-rate independence*: In a typical clinical dose distribution both the accumulated absorbed dose and the dose rate are not uniquely correlated. In most clinical dose distributions, the dose rate in one location varies during the treatment. If the dose response was dependent on the dose rate, a similar dose delivered at a different dose rate would result in a different dose reading. It is important to note that even for a single beam, the dose rate varies significantly in depth and in the penumbra region.
- *Energy independence*: Most linacs deliver photon radiation beams, of which the photon energy covers a large range of energies. Three dimensional dosimeters can also be employed for dosimetry of orthovoltage, brachytherapy and electron radiation. It is, therefore, desirable that the dosimeter is energy independent.
- *Linear energy transfer (LET) independence*: LET independence is especially important for particle therapy. In particle beams, the LET varies which reaches a maximum near the end of the Bragg peak. The high LET in the Bragg peak makes it challenging for chemical dosimetry as recombination effects often lead to an underestimation of the dose.
- *Long shelf life*: While a long shelf life is no ultimate requirement, the ability to store 3D dosimeters in a clinical medical physics unit for when they are needed makes them more attractive in a clinical setting. Factors that affect the dose response of chemical 3D dosimeters are related to chemical decomposition, auto-oxidation, thermal reactions between chemical components and evaporation of chemicals from the dosimeter. For commercial off-the-shelf 3D dosimeters, it is also important to consider any effects of environmental fluctuations (temperature, pressure, exposure to light) during transport on the dose response.

While some early papers in the first half of the 20th century already mentioned the potential of using chemical dosimeters [3], gel systems [4,5] and polymers [6,7,8] for radiation dosimetry, the field of research into gel systems for 3D radiation dosimetry emerged when a research team at Yale university discovered that the radiation-induced oxidation of ferrous (Fe^2+^) ions in ferrous sulfate dosimeters, initially developed by Fricke and Morse [3], could be measured with NMR relaxometry [9], and attempts to fixate the dose information spatially were undertaken by dispersing the Fricke solution in an agarose gel [10]. A major obstacle for the use of Fricke gel dosimeters in radiotherapy is the diffusion of the ferrous (Fe^2+^) and ferric (Fe^3+^) ions in the gel matrix. Many studies have focused on reducing the diffusivity of the ions by using different hydrogel materials and by using chelators. While some methods were able to reduce the diffusivity, none of the methods were able to completely stop the diffusion.

In 1993, the Yale group also suggested the use of hydrogels immersed in vinyl monomers as potential dosimeters [11]. When exposed to ionizing radiation, radiation-induced water radicals initiate polymerization of the monomers, which results in the formation of small polymer aggregates that become entangled with the hydrogel matrix. The drastic change in polymer mobility has a significant effect on the NMR transverse relaxation rate of the water molecules. As a result, the regions containing polymer aggregates are visible on T_2_-weighted MRI scans. The amount of polymer and, hence, absorbed radiation dose, is displayed quantitatively in MRI R_2_ maps, where R_2_ is the transverse NMR relaxation rate (=1/T_2_). Dose maps can then be obtained by use of a calibration plot of R_2_ versus the radiation dose. While the first dose maps acquired with Fricke gel dosimeters and polymer gel dosimeters demonstrated the potential of 3D dosimetry, gel dosimetry in the early days lacked robustness in many aspects. As the spatial integrity of polymer gel dosimeters appeared superior to that of Fricke gel dosimeters, the interest in Fricke gel dosimeters declined over the years.

The challenge of increasing the accuracy and precision of 3D gel dosimetry led to an active field of multi-disciplinary research that involved contributions from chemists, material scientists, medical physicists, engineers and MRI physicists. Scholars in the field gathered at biennial international conferences under the name “DOSGEL” (1999–2008) and “IC3Ddose” (from 2010) to exchange ideas and collaborate. The conference proceedings are a valuable source of information on the topic and are comprised of proffered papers and elaborate review papers. Since 2004, the conference proceedings have been published by the Institute of Physics [12,13,14,15,16,17,18,19,20]. A comprehensive review paper on the topic of polymer gel dosimetry was also published by the journal Physics in Medicine and Biology [21] and in the form of book chapters [2,22].

The accuracy and precision of polymer gel dosimetry has improved considerably over the years [23,24,25] as the physico-chemical mechanisms of gel systems become better understood [23,26,27,28,29,30,31,32,33,34,35,36,37,38,39,40,41,42], the radiation properties of gel dosimeters characterized [43,44,45,46,47,48,49,50,51,52,53,54,55,56,57,58], the NMR contrast mechanisms described [59,60,61,62], imaging artifacts in quantitative MRI compensated [63,64,65,66,67,68,69,70,71,72,73] and MRI pulse sequences optimized [74,75,76]. However, it is important to remain vigilant about all sources of uncertainty in order to obtain reliable dose maps [23,77]. In addition to the scientific quest for a better understanding of gel dosimeters, several different gel dosimeters have been proposed and other readout techniques have been proposed, such as X-ray CT [78,79,80], optical CT [81,82,83,84,85,86,87] and ultrasonic imaging [88].

To decrease the uncertainty in tumor location during radiotherapy, a series of image guidance techniques were introduced to assess the position of the tumor at the start of treatment or during radiation delivery [89,90]. A recent development in image guided radiotherapy (IGRT) is the introduction of the MR-Linac, which is the hybridization of a linear accelerator and an MRI scanner. The first prototype MR-Linac was developed and installed at UMC Utrecht in 2008 [91], and the first patient was treated in 2017 [92]. Recently, the potential of polymer gel dosimetry for inline radiation dosimetry on an MR-Linac was demonstrated, where dose maps were acquired with the MRI subunit during radiation delivery [93].

This review paper aims to provide an overview of the methods and state-of-the-art technology of 3D radiation dosimetry with gel and polymer systems. The reader is also referred to other review-type papers on gel dosimetry with a stronger emphasis on PVA Fricke gel dosimeters [94] and on low-energy radiation dosimetry [95]. Another recent review-paper in this journal covers hydrogel-based non-3D radiation sensors [96] and the use of polymers in a variety of analytical medical applications [97].

## 2. Fricke Gel Dosimeters

### 2.1. Fricke Solutions

Hugo Fricke reported in 1927 that ferrous ions (Fe^2+^) in a 0.8 N sulfuric acid solution are converted to ferric ions (Fe^3+^) upon irradiation with X-rays [3]. The concentration of Fe^3+^ ions was measured by an electrometric titration. Absorption spectrophotometry was later used to determine the conversion as a selective absorption peak of Fe^3+^ occurs at λ = 304 nm [98,99]. An astonishing observation was that the amount of radiation-induced oxidation was independent of the concentration of ferrous ions if sufficient ferrous ions were present in the solution. It led Fricke to conclude that the absorbed radiation energy converted the water to a chemically activated form that diffused through the liquid and reacted with the dissolved ions. This important finding was later extended to X-ray-induced chemical reactions of organic compounds [100]. Given the abundancy of water in biological cells, the properties of activated water would also prove very important in determining how X-rays affect the processes of life. For decades, Fricke’s ferrous sulfate dosimeters have served as a dosimetry standard in all laboratories where relatively high intensity X-rays or gamma-rays were used [101]. Because of the strong dependence of the radiochemical yield (G) on the linear energy transfer (LET) of the radiation [102], Fricke solution has fallen into disuse.

The conversion of Fe^2+^-ions to Fe^3+^-ions by ionizing radiation also alters the magnetic moment and electron spin relaxation times of the metal ion. These changes result in a change in NMR relaxation rates. In 1984, it was demonstrated that the change in magnetic moment and electron spin moment resulted in a change of the transverse relaxation rate with a radiation dose of 1.13 × 10^−2^ s^−1^ Gy^−1^ and a similar change in transverse relaxation rate of 1.21 ± 0.27 × 10^−2^ s^−1^ Gy^−1^ [9]. It was also suggested that infusion of a hydrogel with the Fricke solution could result in a 3D dosimeter.

### 2.2. Radiation Chemistry and Chemical Yield

The NMR dose sensitivity of a Fricke solution can be calculated theoretically from the radiochemical yield (i.e., the radiation-induced change in the molar concentration of iron ions with absorbed dose) and from the relaxivity of Fe^2+^ and Fe^3+^ ions. The radiochemical yield (G) can be calculated on the basis of the radiation chemistry of the Fricke solution.

**Figure 1:**
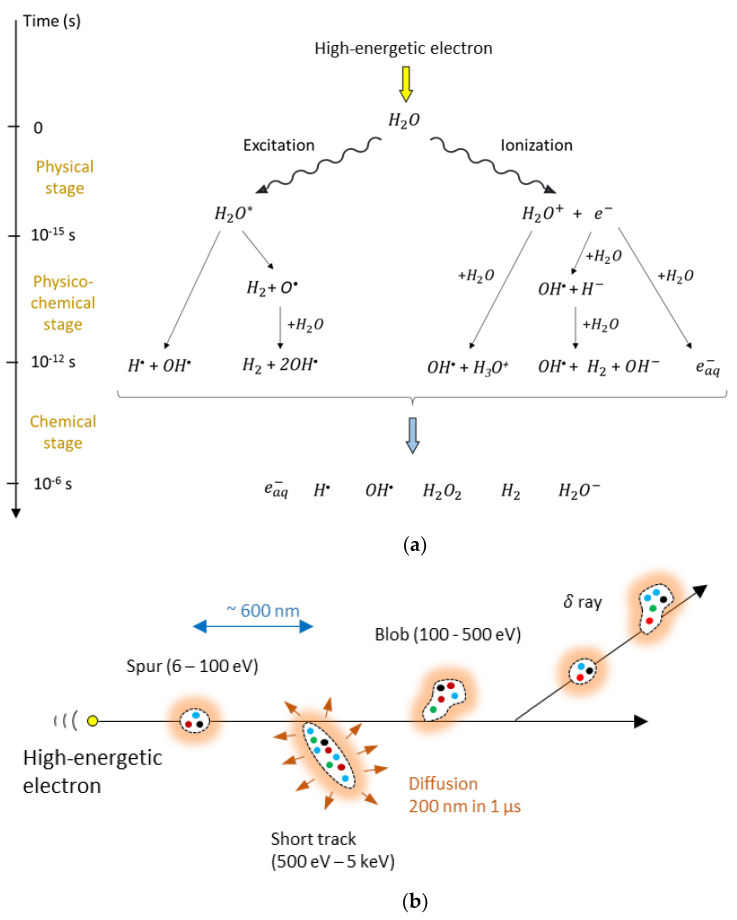
High-kinetic energy electrons create radiolytic products of water in what is described as a three-stage process (**a**). At the end of the radiolytic process, six radiolytic species can be considered. The distribution after 1 µs is shown in (**b**). The colored dots represent the six different radiolytic species that are grouped in spurs, short tracks and blobs. The radiolytic species diffuse further and, in a Fricke dosimeter, react with iron ions.

When high-energetic photons penetrate in a Fricke solution, the high-energetic photons do not immediately ionize the ferric ions in a single step. Instead, the high-energetic photons undergo scattering by interactions with electrons predominantly from water. Textbook radiation physics teach that these interactions occur in the form of the photo-electric effect, Compton scattering or with involvement of the nucleus if the photon has an energy in excess of 1.022 MeV, which results in the creation of a positive and negative electron pair. The interaction of ionizing radiation with molecules is governed by the transfer of energy from the high-kinetic energy electrons created by the scattering processes. The high-kinetic energy electrons undergo small ionization losses by coulombic interaction with the bound electrons of the medium. This multiple collision process is the predominant mechanism in the deposition of energy in the molecules of the medium. The density of energy absorbed in the medium per unit mass is the absorbed dose (*D*).

(1) $$D=\frac{dE_{ab}}{dm}$$

where *E_ab_* is the absorbed dose and *dm* is an elementary amount of mass. Important to note is that the energy is deposited in the medium in a rather erratic manner as a result of the stochastic nature of the interactions, so dose is only meaningful when the mass Δm in which the absorbed energy is considered is sufficiently large to average out any statistical variations. High-kinetic energy electrons transfer energy into molecules by ionization of the molecule, exciting the energy of an electron from a ground state to a higher-energy state (excitation) or to an energy greater than the ionization energy (superexcitation) [103]. The deposition of energy along the track of the high-kinetic energy electron is not distributed uniformly but can be modeled by a “string of beads” model [104] and is illustrated in Figure 1.

The time evolution of the radiolytic process can be described by a three–stage process [105] as illustrated in Figure 1a, while the spatial distribution is illustrated in Figure 1b. Radiolytic yields of the primary radiolytic products of pure water at neutral pH and of water in a 0.8 N sulfuric acid aqueous solution for Cobalt-60 γ-radiation at the end of the chemical stage are provided in Table 1 [106]. The yields depend on several parameters, such as the kind of radiation (i.e., the linear energy transfer, LET and the acidity (pH)).

The radiolytic water products initiate the oxidation of ferrous ions in a set of reactions, as outlined in Equations (2)–(7).

(2) $$H^{•}+O_{2}\to {\;\mathrm{HO}}_{2}^{•}$$

(3) $${\mathrm{HO}}_{2}^{•}+{\mathrm{Fe}}^{2+}\to {\;\mathrm{HO}}_{2}^{-}+{\mathrm{Fe}}^{3+}$$

(4) $${\mathrm{HO}}_{2}^{-}+H^{+}\to H_{2}O_{2}$$

(5) $${\mathrm{OH}}^{•}+{\mathrm{Fe}}^{2+}\to {\mathrm{OH}}^{-}+{\mathrm{Fe}}^{3+}$$

(6) $$H_{2}O_{2}+{\mathrm{Fe}}^{2+}\to {\mathrm{OH}}^{-}+{\mathrm{Fe}}^{3+}+{\mathrm{OH}}^{•}$$

(7) $$H^{•}+H_{2}O\to {\;\mathrm{OH}}^{•}+H_{2}$$

It can be noted that each primary radiolytic hydrogen radical H^•^ results in the oxidation of three Fe^2+^ ions. Indeed, as can be seen from Equation (2), the reaction of H^•^ with oxygen results in the creation of HO_2_^•^ which oxidizes a first ferrous ion and is thereby reduced to HO_2_^−^ (Equation (3)). The HO_2_^−^ created in Equation (3) further reacts with H^+^ to form hydrogen peroxide (4), which then oxidizes a second Fe^2+^ ion (6). The initial hydrogen radical can also react with a water molecule to create a hydroxyl radical OH^•^ (7), which can then oxidize a third ferrous ion (5). As a result, a single hydrogen radical oxidizes three ferrous ions. The radiolytic hydrogen peroxide H_2_O_2_ oxidizes two ferrous ions, one directly (6) and one indirectly by the production of the hydroxyl radical in (6) that oxidizes a second ferrous ion (5). The initial radiolytic hydroxyl radical OH^•^ oxidizes a single ferrous ion (5). As a result, the theoretical chemical yield of the ferrous/ferric ion conversion can be summarized as a function of the chemical yield of the radiolytic water radicals:

(8) G(Fe^3+^) = 3 G(H^•^) + 2 G(H_2_O_2_) + G(OH^•^)

Filling in the values of the radiolytic gain of primary water products for a standard Fricke solution with 0.8 N H_2_SO_4_ (Table 1, 3rd column) in Equation (8) gives the absolute theoretical chemical yield of the Fricke dosimeter and is about 15.6 Fe^3+^ ions per 100 eV of imparted radiation energy, or 1.61 µM/Gy (using Table 1, 4th column).

### 2.3. MRI Contrast Mechanism and Dose Sensitivity

**Figure 2:**
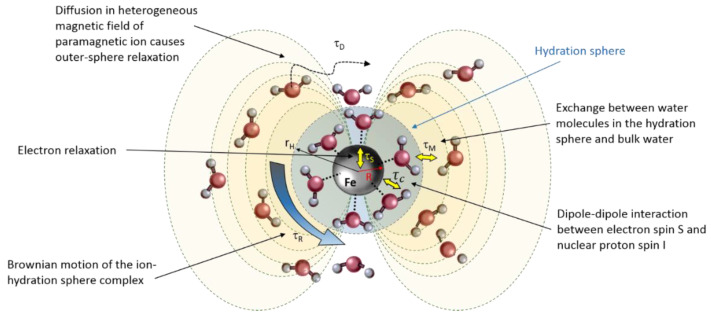
Schematic representation of the different contributions to the NMR relaxation caused by the paramagnetic Fe^2+^ and Fe^3+^ ions. τ_S_: electron relaxation time, τ_c_: correlation time of dipole–dipole interaction between electron spin S and proton nuclear spin I, τ_M_: residence time, τ_R_: rotational correlation time, τ_D_: water diffusion correlation time. r_H_ is the radius of the hydration sphere.

In aqueous solutions, the longitudinal (T_1_) and transverse (T_2_) proton relaxation times are determined by the magnetic field fluctuations experienced by each hydrogen proton (^1^H) nucleus, which originate from the molecular motions inside the liquid [107]. Both ferrous (Fe^2+^) and ferric (Fe^3+^) ions are paramagnetic and influence the proton relaxation rate of water molecules that reside in the vicinity of the ion significantly. The magnetic moment of such paramagnetic ions has dipolar interaction energies that are about 659 times larger than the proton magnetic moment. The paramagnetic ions are surrounded by several water molecules depending on the ionic radius. The sphere of surrounding water molecules is referred to as the hydration sphere. An overview of the different interactions is given in the schematic drawing of Figure 2.

The longitudinal and transverse relaxation is described by the Solomon-Bloembergen-Morgan equations [108], modified by Connick and Fiat [109], and are composed of contributions from:

(1) dipole–dipole interactions between the ion electron spin S and proton nuclear spin I, characterized by a correlation time τ_c,_ which by itself is constituted of the three temporal magnetic field modulation times (τ_R_, τ_M_ and τ_S_) (see Figure 2). The dipole–dipole interaction term is a short-range interaction that is inversely proportional to the sixth power of the distance between the center of the ion and the water hydrogen protons.
(2) Another interaction term involves the weaker scalar coupling. The scalar coupling correlation time contains both the residence time τ_M_ and electron relaxation time τ_S_.
(3) A third contribution comes from the water molecules in the outer coordination sphere that experience a diffusion weighting with diffusion correlation time $\tau_{D}=d^{2}/\left(D_{I}+D_{S}\right),$ where d is the closest distance of approach between the ion and the hydrogen aFtom, $D_{I}$ is the diffusion coefficient of the water molecule and $D_{S}$ is the diffusion coefficient of the ion.

For a full quantitative description of the different interaction terms, the reader is referred to [22]. In the fast diffusive exchange regime, the relaxation of the different proton pools averages out physically, resulting in a mono-exponential relaxation with relaxation rate $R_{k}$ (k = 1, 2):

(9) $$R_{k}=f_{b}\left(R_{k,\mathrm{is}}+R_{k,\mathrm{os}}\right)+\left(1-f_{b}\right)R_{k,0}\;(k=1,\;2)$$

where $R_{k,\mathrm{is}}$ is the (longitudinal (R_1_) or transverse (R_2_)) relaxation rate of the water molecules in the inner (hydration) sphere, $R_{k,\mathrm{os}}$ is the relaxation rate of the water molecules in the outer coordination sphere, $R_{k,0}$ is the relaxation rate in bulk water and $f_{b}$ is the fraction of protons in the hydration sphere that is proportional to the concentration of ions *C* (i.e., $f_{b}=\kappa C$). The proportionality constant is determined by the number of water molecules in the hydration sphere and amounts to $\kappa =0.108{\;M}^{-1}$. As the outer sphere contribution can be ignored, the relaxation rate for a Fricke solution containing both ions can be written as:

(10) $$R_{k}≅\kappa \left[{\mathrm{Fe}}^{2+}\right]R_{k,\mathrm{is}}^{{\mathrm{Fe}}^{2+}}+\kappa \left[{\mathrm{Fe}}^{3+}\right]R_{k,\mathrm{is}}^{{\mathrm{Fe}}^{3+}}+R_{k,0}\cdot (k\;=\;1,2)$$

The relaxation after an absorbed radiation dose D then becomes:

(11) $$R_{k}=R_{k,0}+\kappa {\left[{\mathrm{Fe}}^{2+}\right]}_{0}R_{k,\mathrm{is}}^{\mathrm{Fe}2+}+\kappa \left(\frac{\partial \left({\mathrm{Fe}}^{3+}\right]}{\partial D}\right)\left(R_{k,\mathrm{is}}^{\mathrm{Fe}3+}-R_{k,\mathrm{is}}^{\mathrm{Fe}2+}\right)\cdot D$$

where $\frac{\partial \left({\mathrm{Fe}}^{3+}\right]}{\partial D}$ is the aforementioned theoretical radiation yield (1.61 µM/Gy). The relaxation dose sensitivity is described by the factor in the third term:

(12) $$r_{k}^{D}=\kappa \left(\frac{\partial \left({\mathrm{Fe}}^{3+}\right]}{\partial D}\right)\left(R_{k,\mathrm{is}}^{\mathrm{Fe}3+}-R_{k,\mathrm{is}}^{\mathrm{Fe}2+}\right)$$

After filling in the values in Equation (11), we find for a field strength of 0.47 T, $r_{1}^{D}=0.013{\;s}^{-1}{\mathrm{Gy}}^{-1}$ and $r_{2}^{D}=0.0178{\;s}^{-1}{\mathrm{Gy}}^{-1}$. These values correspond very well with measured values by Gore et al.: $r_{1}^{D}=0.0113{\;s}^{-1}{\mathrm{Gy}}^{-1}$ and $r_{2}^{D}=0.0121s^{-1}{\mathrm{Gy}}^{-1}$ [9]. Because the dipole–dipole interaction is dependent on the magnetic field (Larmor frequency), the relaxation rates are also field (frequency) dependent.

In Fricke gel dosimeters, the Fricke solution is dissolved in a hydrogel matrix. Both agarose and gelatine were used as gelling agents. In a Fricke gel in the fast exchange regime, the relaxation rate can be expressed as

(13) $$R_{k}=R_{k,0}+r_{k}^{{\mathrm{Fe}}^{2+}}{\left({\mathrm{Fe}}^{2+}\right]}_{0}+r_{k}^{\mathrm{gel}}\left(\mathrm{gel}\right]+r_{k}^{D}D\;\;\;\;\left(k=1,2\right)$$

where $R_{k,0}$ is the relaxation in free water, $r_{k}^{{\mathrm{Fe}}^{2+}}$ is the relaxivity of ferrous ions in units of s^−1^.M^−1^, ${\left({\mathrm{Fe}}^{2+}\right]}_{0}$ is the initial concentration of ferrous ions (ferrous sulphate), $r_{k}^{\mathrm{gel}}$ is the relaxivity of the gel and $r_{k}^{D}$ is the relaxation dose sensitivity.

As sulfuric acid degrades the gel matrix, lower concentrations of sulfuric acid (0.05 M) are typically used, and care is taken to only mix the sulfuric acid solution into the gel mixture at temperatures as low as possible before the gel starts to solidify. Concentrations of sulfuric acid higher than 50 mM generally result in poor uniformity of the dosimeter, which is attributed to a temperature dependent degradation of the gel matrix by the acid. Moreover, a higher concentration of sulfuric acid was not found to result in a significant increase in dose sensitivity [110]. It was found that the radiochemical yield was higher in Fricke gel dosimeters than in Fricke solutions [10], with a 4-fold increase in the R_1_-dose sensitivity in a 1% (*w*/*w*) agarose Fricke gel dosimeter and an increase in dose-sensitivity with a factor of 2.2 in a 4% (*w*/*w*) gelatine Fricke gel dosimeter [111]. A further increase in agarose concentrations did not result in an increase in dose sensitivity and even a moderate decrease was observed for gelatine concentrations in the range of 4–12% [112]. This decrease was attributed to an increase in pH, which results in the formation of ferric ion hydroxide complexes, which reduces the number of hydrogen protons in the hydration sphere. Other additives were also investigated, such as benzoic acid, xylenol orange and salt. Benzoic acid was added in an attempt to increase the dose sensitivity, as earlier studies showed an increase in dose sensitivity in Fricke solutions. However, it was found that the addition of benzoic acid in gelatine Fricke gel dosimeters did not change the dose response [113]. Xylenol orange can be used as an optical indicator, opening the possibility for Fricke gel dosimeters to be scanned with optical CT [114], but the addition of xylenol orange decreased the R_1_-dose sensitivity [115], which was attributed to a chelation of the ions, which shields the water molecules from the ion. An increase in both R_1_ and optical density sensitivity was observed when polyvinyl alcohol gel (PVA) dosimeters were doped with sucrose but remained lower than that of an undoped agarose-based Fricke gel [116]. As oxygen is found to increase the sensitivity, Fricke gels can also be purged with oxygen gas [111]. In order to obtain a linear R_1_-dose response in an agarose Fricke gel, purging with oxygen was found necessary [111] to compensate for the loss of oxygen during heating of the agarose gel at high temperatures. The addition of salt was found to decrease the R_2_-dose sensitivity [117]. Table 2 provides an overview of studies on the effect of chemical and radiation factors on the dose response of Fricke gel dosimeters.

No significant dose-rate dependence was found for Fricke agarose gel dosimeters in the range of 1 Gy/min–24.2 Gy/min [110,112]. The electron density and effective atomic number are measures of the tissue equivalence of the dosimeter and can be calculated from the stoichiometric composition of the gels [122]. Both agarose and gelatine-based Fricke gel dosimeters can be considered as tissue equivalent dosimeters with effective atomic numbers Z_eff_ = 7.46 and 7.56, respectively, where water has an effective atomic number Z_eff_ = 7.42. The electron densities relative to water are 1.006 and 1.004 for agarose and gelatine-based gel dosimeters, respectively. Moreover, no significant dependence on radiation quality was found for Fricke gel dosimeters in the range from 6 MeV to 18 MeV [110,112].

It was soon discovered that the dose distribution registered in Fricke gels lacked spatial integrity as a result of the diffusion of ferric and ferrous ions in the gel dosimeter. The diffusion coefficient of an agarose-based gel dosimeter is in the order of 2.3 × 10^−10^ m^2^/s. Many attempts were made to change the composition of the gel to decrease the diffusion of the paramagnetic ions. Approaches to measure the diffusion coefficient are discussed in [22]. Two strategies were explored to decrease the diffusion coefficient: The first strategy involves the addition of a chelating agent with the intention to decrease the mobility of the ion. Xylenol orange halves the diffusion coefficient but at the cost of a significant decrease in dose sensitivity. A second strategy involves changing the morphology of the gel in a way that restricts the mobility of the ions through the maze-like structure of the gel matrix. The diffusion coefficient of the iron ions in agar gel is higher than in gelatine gel. A two-fold reduction in the diffusion coefficient is achieved in a combined 1.5% agarose/3% gelatine gel [124]. One of the smallest ion diffusion coefficients was found in a poly-vinyl-alcohol (PVA) Fricke gel at 3.9 × 10^−11^ m^2^ s^−1^ in a 20% PVA gel [125], a reduction with a factor 6 as compared to a standard agarose Fricke gel. PVA gel is fabricated with a cycle of freezing and thawing. The viscosity of a PVA gel increases with every freeze–thaw cycle, which is attributed to an increase in cross-linking which also decreases the relaxation time. Another way of creating a PVA gel is by use of chemical cross-linking with, for example, glutaraldehyde (GTA) [126]. The two aldehyde groups on the GTA react with hydroxyl-groups on the PVA creating acetal bridges [127]. PVA-GTA Fricke gels have an R_1_-dose sensitivity around 0.025 s^−1^.Gy^−1^, approximately double that of standard agarose Fricke gels independent of the temperature during irradiation [128]. The R_1_-dose sensitivity increases further with another factor of 3 after addition of xylenol orange [129,130,131]. No significant changes in the dose sensitivity or ion diffusion coefficient were found for xylenol orange PVA-GTA Fricke gels prepared at different gelation temperatures between 6 °C and 42 °C. As in other Fricke gel systems, auto-oxidation appears to result in instability of the dose–response curve, which is accelerated at higher storage temperatures [124]. Lower auto-oxidation rates were found in PVA-GTA Fricke gel dosimeters but the purity of the PVA is critical [132]. Only a minimal change in optical dose sensitivity with dose rate was detected in the dose-rate interval [70 cGy.min^−1^, 348 cGy.min^−1^] [128]. The optimal concentration of ferrous ammonium sulphate and xylenol orange are found to be in the range of 0.4–1 mM and 0.166–0.2 mM, respectively [133]. Chelating agents other than xylenol orange were investigated, such as methylthymol blue (MTB) [133,134,135] and 5-sulfosalicylic acid as a replacement for sulfuric acid [136]. The addition of the free radical scavenger dimethyl sulfoxide to a MTB-PVA-GTA Fricke gel dosimeter results in a reduction in the diffusion coefficient to 2.1 × 10^−11^ m^2^ s^−1^ [137].

Strategies to further reduce the diffusion coefficient could be focused on anionic hydrogels, the addition of viscosity-increasing agents, the dispersion of ferrous ions in liposomes, the use of sorbent polymers and functionalization of the hydrogel backbone with complex forming groups. Challenges that come with these strategies are that the radiation-induced oxidation reaction should not be compromised, that the gel is tissue-equivalent, that there is no significant loss in dose sensitivity, that the gel dosimeter is dose-rate independent and that the resulting dosimeter remains affordable. The ion diffusion is a big limitation in Fricke gel dosimeters, which explains the limited dissemination of these 3D dosimeters in clinical practice.

### 2.4. Applications of Fricke Gel Dosimeters

Many contemporary radiation treatment modalities use dynamic techniques where many beams are delivered over different time spans. In these treatments, both the absorbed dose and the dose rate vary from point-to-point in the patient. Because of the variation in the time evolution of dose deposition in each point, it is difficult to obtain reliable dose measurements with instantaneous point-detectors such as ion chambers or diode detectors. Integrating chemical dosimeters such as Fricke gel dosimeters has the potential to acquire the integrated dose distribution in three dimensions. A basic case of a dynamic treatment is a wedged field delivered with dynamic wedge photon beams. In a wedged field delivered to a flat phantom, the isodose lines make an inclination with the surface of the phantom, of which the inclination angle is the wedge angle. The wedged field is created by moving one of the field-defining jaws in the collimator head gradually across the beam. Relative dose distributions obtained with Fricke gel dosimeters irradiated with a dynamic edge technique showed good agreement with the nominal values and alternative dose measurements with a linear diode array and treatment planning, but a 5% lower dose was registered with Fricke gel near the field edges [138]. The 5% dose deviation near the beam edges is attributed to ion diffusion in the 4% (*w*/*w*) gelatine Fricke dosimeter.

Fricke gel dosimeters were applied to validate the treatment plans for clinical multi-beam treatments for bladder and breast cancer [139]. In the breast treatment, a photon beam was combined with two electron beams. While the dosimetry proved that both treatments satisfied clinical acceptance criteria, small deviations between the Fricke gel measured dose distributions and the calculated dose from the treatment plan were attributed to field adjustment errors, partial volume effects in the dose calculations and an underestimation of electron scatter from the electron applicator in the dose calculations. The uncertainty in relative dosimetry using Fricke gel dosimeters was significantly improved to approximately 2% for single beam dose distributions by optimizing the scanning protocol and applying spatial filtering to increase the SNR of the images [140,141]. Higher uncertainties were found in 3D conformal radiotherapy dose verifications, which were attributed to variations in the environmental temperature during the experiment [142].

Anthropomorphic Fricke gel dosimeters with lung-equivalent low-density Fricke gel were proposed for dose verification of lung cancer intensity modulated radiotherapy (IMRT) treatments [143]. The low-density Fricke gel dosimeter was obtained by dispersing Styrofoam beads in the gel. A change in dose sensitivity in the lung equivalent dosimeters between 0.8 and 1.55 was found [144].

Fricke gel dosimeters were also applied to determine the dose distribution of intra-cavitary brachytherapy using radioactive 192-Iridium or 106-Ruthenium sources [145,146], but care is required in evaluating the dose close to the brachytherapy source where high dose gradients may be obscured by ion diffusion.

Another challenging application for Fricke gel dosimetry is stereotactic radiosurgery where high doses (40 Gy) are delivered to small regions of the brain. Typical applications of stereotactic radiosurgery are gliomas or metastatic brain tumors and arterial–venous malformations (AVMs). As a high dose is delivered in a single fraction, it is critical that the dose is delivered precisely in the target volume. Therefore, a stereotactic head frame is used to position the patient uniquely with respect to the radiation beams. Validation of stereotactic radiotherapy with Fricke gel dosimeters was proposed, but it was concluded that the dose precision of the dosimeter was not sufficient to be used as a basic dosimeter for the gamma knife [147,148].

The emergence of proton therapy facilities and intensity modulated proton therapy (IMPT) has also increased the need for integrating 3D dosimeters. Despite the significantly higher cost of proton therapy, treatment with protons and heavy ions have specific advantages associated with the physical properties of the high energetic particles. Protons and heavy ions exhibit a high energy deposition density close to the end of the spatial range of the particles and result in limited lateral scatter. The spatial range of the particles can be modulated by changing the kinetic energy of the particles. These properties enable the delivery of beams with larger dose gradients. The R_1_-dose sensitivity of Fricke gel dosimeters for proton beams at a mean proton energy of 90 MeV was found to be similar to that for high-energetic photons, but the relative R_1_ in the Bragg peak was found to underestimate the dose with 15–20% [149]. The dependence of the dose sensitivity on the linear energy transfer (LET) is attributed to a recombination of water radicals at high LET. An additional factor that could be responsible for an underestimation of R_1_ in the Bragg peak is ion diffusion. An LET independent Fricke gel dosimeter was proposed more recently [150]. Here, a nanoclay (Laponite XLG) was added to a gelatine Fricke gel dosimeter, and sulfuric acid was replaced by perchloric acid. The nanocomposite Fricke gels were irradiated with carbon and argon ion beams covering an LET range of 10 to 3000 eV/nm. The precise mechanism of the LET independence in these systems is still unknown. While the first applications of Fricke gel dosimeters demonstrated great potential in verifying radiation dose distributions, the lack of spatial integrity over time has avoided further dissemination of these radiation dosimeters in radiotherapy practice.

When Fricke gel is doped with a color indicator such as xylenol orange, a dose dependent optical attenuation at particular wavelengths occurs. The optical absorbance is proportional to the absorbed radiation dose. It is important that the gel does not scatter the light. While agarose-based Fricke gel dosimeters were scanned optically, the accuracy is compromised because of light scattering. An optically transparent gel matrix is preferred, such as gelatine or Pluronic F-127. It was suggested that gelatine-based Fricke gel dosimeters doped with xylenol orange, referred to as FXG gel dosimeters, have practical clinical use when combined with fast optical readout after exposure [151,152,153,154].

Fricke gel dosimeters were also applied in commissioning in an MR-Linac [155]. The large magnetic field causes a Lorentz force on the secondary electrons that are released in matter during the irradiation with high-energetic photon beams. This may result in significant alterations of the dose distribution with respect to that found on conventional Linacs (in the Earth’s magnetic field). Such effects are clearly visible near transitions between high-density and low-density tissues such as the lungs. Electrons entering a low-density tissue may be re-directed toward the high-density region by the Lorentz force, resulting in hot spots of the dose in the high-density region. This, so-called electron return effect was observed by use of a two-compartment FXG gel, where a lower density was obtained by use of polystyrene beads [156]. While some signal change in Fricke gel dosimeters was visually observed during radiation [157], the temporal uncertainty is too large for quantifiable real-time 4D dosimetry with inline MRI readout [93].

## 3. Radiochromic Gel Dosimeters

Other gel systems that exhibit a color change upon irradiation were proposed. Similar to the FXG gel dosimeter, these radiochromic gel dosimeters can be read out by the use of optical CT scanning.

### 3.1. Micelle Gel Dosimeters

Micelle gel dosimeters are hydrogels in which micelles are dispersed that contain a radiation sensitive hydrophobic dye, such as leuco-malachite green or leuco-crystal violet, and an organic halogen, such as chloroform or trichloroacetic acid, that acts as an initiator. To stabilize the micelles in the hydrogel, a surfactant, such as Triton-X or sodium-dodecyl-sulphate (SDS), is used [158,159]. Because the leucodye is more soluble in the organic phase of the micelles than in the surrounding hydrogel, the spatial integrity of the micelle gel dosimeter is significantly better than that of Fricke gel dosimeters. With some formulations of micelle gel dosimeters, a significant dose-rate dependence was found [160], while other formulations appear to be dose-rate independent over the range of 100 to 600 cGy.min^−1^ [161]. The micelle gel dosimeter was applied for small-field dosimetry [162] and clinical dose verification of a pituitary gland tumor with comparable results to polymer gel dosimeters [163]. A micelle gel dosimeter was also irradiated with carbon ions [164]. While the measured dose distribution does not match with the expected dose distribution derived with Monte Carlo simulations, it is suggested that the response of the micelle gel dosimeter is related to the creation of OH-radicals.

### 3.2. Turnbull-Blue Gel Dosimeters

The Turnbull-blue gel dosimeter [165,166] is composed of a Phytagel™, active ingredients potassium ferricyanide (K_3_Fe(CN)_6_), ferric chloride (FeCl_3_·6H_2_O) and ferric ammonium citrate (C_6_H_8_O_7_·FeNH_3_), that, upon irradiation, forms the dye Turnbull-blue, K[Fe^II^Fe^III^(CN)_6_. The Turnbull-blue dye is nearly insoluble in water and forms micelles that cannot easily diffuse through the gel matrix but are small enough to not render the gel opaque. The diffusion coefficient of the Turnbull-blue micelles is at least two orders of magnitude smaller than that of FXG gel dosimeters. The Turnbull-blue gel dosimeter was applied to measure relative output factors of a gamma knife treatment system with good correspondence with treatment planning [167,168].

### 3.3. TruView™ and ClearView™

Two radiochromic gel dosimeters were commercialized by the company Modus QA (Modus Medical Devices Inc., London, ON, Canada). The ClearView™ dosimeter is based on the colorless redox indicator tetrazolium salt, which converts to an insoluble formazan dye, which is purple, upon radiation [169]. The insoluble formazan dye is spatially stabilized by the gel matrix, which can consist of either gelatine or gellan gum. Practical experience with the use of a commercial ClearView™ dosimeter inserted in a head phantom for the validation of a stereotactic radiosurgery treatment was reported [170]. The use of a physical gel matrix Pluronic F-127 was suggested as an alternative to gelatine or gellan gum. In these gel dosimeters, the color turns to red upon irradiation [171].

The second gel dosimeter is based on a Fricke gel dosimeter with Methylthymol blue (MTB) as an indicator and is trade-named TruView™. The TruView™ gel dosimeter is found to be more sensitive than FXG Gel dosimeters [172]. The maximum spectral change occurs at a wavelength of 632 nm for the TruView™ gel dosimeter and at 530 nm for the ClearView™ gel dosimeter [173]. The optimal time of readout of the TruView™ gel dosimeter is set at 90 min after irradiation, which is a compromise between chemical instability and loss of spatial integrity as a result of ion diffusion. The ClearView gel dosimeter is found to be more stable but has a far lower dose sensitivity. Until now, both dosimeters were only used with a relative dose calibration.

### 3.4. PVA–Iodide Gel Dosimeters

Recently, radiochromic gel dosimeters have been proposed that are based on the complexation of polyvinyl alcohol (PVA) and iodide [174]. Upon irradiation, radiolytic water products initiates the formation of tri-iodide ions that form a complex with the PVA molecules. Aside from favorable properties such as a minimal dose-rate dependence and high spatial stability, these gel dosimeters demonstrate the promising characteristic that they can be reset by heating the gel to 45 degrees Celsius for 24 h [175]. Some diffusion of tri-iodide ions was observed, but the diffusion coefficient (2 × 10^−12^ m^2^ s^−1^) is an order of magnitude smaller than the diffusion of ferric ions in MTB-PVA-GTA Fricke gel dosimeters. PVA–Iodide dosimeters are water-equivalent for high energy photon beams (>200 keV), but the dosimeters demonstrate a characteristic K-edge photon absorption peak at 33.2 keV, which increases with increasing potassium iodide concentration.

### 3.5. D Plastic and Elastomer Dosimeters

Strictly speaking, radiochromic plastic and elastomer dosimeters are not considered gel systems, but for the sake of completeness, it is worth mentioning polyurethane and silicone-based dosimeters. Polyurethane dosimeters [176] have gained popularity as they are also commercially available under the tradename PRESAGE^®^ (Heuris, Inc., Skillman, NJ, USA). Polyurethane dosimeters are more difficult to fabricate on site because of the use of pressurized vessels in the manufacturing process, but the dosimeters can be machined into different sizes and shipped as an end product. PRESAGE^®^ has a 10% higher mass density than water and a higher effective atomic number (Z) than water and most hydrogel-based dosimeters. As a result, differences in Compton and photoelectric interaction probability between PRESAGE^®^ and water reached up to 55% and 85%, respectively [56]. This is of particular importance for irradiations involving lower photon energies. PRESAGE^®^ dosimeters also demonstrate a temperature dependence during radiation and storage [177] and some temporal instability [178] making it difficult to use the dosimeter with absolute calibration. Some dependence of the dose sensitivity on oxygen content in PRESAGE^®^ was also found [179]. Despite these unfavorable properties, the relative dose distribution was found to be reproducible within a 2% dose difference for an allowable distance-to-agreement of 2 mm [178], and PRESAGE^®^ was applied in some clinical dosimetric validation studies including IMRT [180,181], VMAT [182,183] in the determination of output factors of gamma knife radiosurgery [184] and in brachytherapy [185,186,187]. PRESAGE^®^ was also tested with hadron therapy, where a 20% dose underestimation was found in the Bragg peak for protons [188], but in another study with 400 MeV carbon ions, no significant LET effect on the dose response was observed [189]. More studies are needed to investigate the dose response of PRESAGE^®^ in hadron therapy. The advantage of chemical 3D dosimeters is that dose measurements are not compromised by large magnetic fields, rendering them suitable for commissioning and QA in MR-Linac systems. The influence of the magnetic field on the dose response of PRESAGE^®^ was studied [190,191] and no significant changes in the dose reading as a result of the magnetic field were detected. The feasibility of applying PRESAGE^®^ in combination with Monte Carlo simulations to validate a commercial magnetic resonance guided intensity modulated radiation therapy (MRgIMRT) system [192] was demonstrated while following a strict protocol to minimize the influence of temporal and spatial changes on the measured dose distribution [193].

A flexible radiochromic dosimeter was also proposed. Here, a silicone elastomer matrix is used in combination with a leucodye and halogen initiator. This system is also known under the name Flexydos3D [194]. While most optical CT is conducted on cylindrical shaped phantoms, some evidence is given that optical CT is not restricted to cylindrical geometries [195]. The Flexydos3D dosimeter has great potential in dosimetric applications in conditions of tissue deformation as the mechanical properties can be tuned [196]. Similar to PRESAGE^®^, no significant effect on the dose response of a magnetic field of 0.35 T was detected for FlexyDos3D [197]. Some dependency of the dose-sensitivity with respect to the dose rate was found in the initial FlexyDos3D dosimeter [194], but it was suggested that by using an optimized chemical formulation the dose-rate effect can be eliminated [198]. Another challenge in the fabrication of FlexyDos3D is to maintain a homogeneous spread of the initiator throughout the phantom [199]. A linear energy transfer (LET) dependent quenching in the proton beam Bragg peak was detected, which can be reduced by increasing the concentration of the curing agent but comes at the cost of a decreased dose-sensitivity [200]. FlexyDos3D has a slightly higher electron density and a 40% higher effective atomic number than water. Monte Carlo simulations show that this has no significant effect on dose distributions of the MV photon beams while corrections would be required at photon energies below 200 keV [201]. Additive manufacturing of FlexyDos3D dosimeter phantoms was recently proposed [202].

### 3.6. Radio-Fluorogenic Dosimeters

Radio-fluorogenic dosimeters (RFLDs) contain components that become fluorescent upon radiation. One type of RFLDs is based on N-(1-pyrenyl)maleimide (MPy), which, upon radiation, co-polymerizes with methyl-methacrylate. As MPy is incorporated in the growing polymer, it becomes fluorescent [203,204,205]. The fluorescence signal is found to be linear with doses up to 1 kGy. A significant dose-rate dependence of the dose sensitivity was found for RFLDs that was inversely proportional to the dose rate [204]. Other radio-fluorogenic gel dosimeters are based on coumarin-3-carboxylic acid. Here, hydroxyl-radicals react with coumarin-3-carboxylic acid, creating 7-hydroxy-coumarin-3-carboxylic acid, which has an emission wavelength at 445 nm upon UV excitation [206]. The gel matrix can be composed of agarose, gelatin [207] or nanoclay [206]. Nanoclay gels have a higher optical transmittance than agarose or gelatin gels and result in a higher dose sensitivity as some inhibition occurs in gelatin gels. To read out RFLDs, a planar excitation light source can be applied that is traversed throughout the irradiated volume [208].

## 4. Polymer Gel Dosimeters

Polymer gel dosimeters (PGDs) are hydrogels in which vinyl monomers are dispersed [21]. Similar to Fricke gel dosimeters, the polymer gel can be poured into a humanoid shaped phantom. From a chemical point-of-view, two different types of polymer gel dosimeters can be considered. In one type, a linear monomer is combined with a large amount of cross-linker monomer. When exposed to ionizing radiation, the monomers undergo a radiation-induced radical chain reaction. This radiation-induced polymerization reaction results in the formation of highly cross-linked microscopically small polymer aggregates that are entangled with the gelatine matrix, which fixates them spatially. Because of the relatively large size of polymer aggregates and the entanglement with the gelatin, the mobility of the hydrogen-bearing functional groups on the polymer is heavily restricted. Typical linear monomers in this type of polymer gel dosimeter are acrylamide, vinylpyrrolidone and poly(N-isopropylacrylamide). A typical cross-linker monomer is N,N′-methylene-bis-acrylamide. Another type of polymer gel dosimeters, further referred to as “acrylic acid-based polymer gel dosimeters”, contains a linear monomer such as methacrylic acid that grafts onto the gel matrix, such as gelatin. No cross-linker monomer is used with this type of polymer gel dosimeters.

The degree of polymerization is related to the absorbed radiation dose. The change in molecular mobility of the polymer has a significant effect on the transverse relaxation. Hydrogen on the hydroxyl groups and amino groups of the polymer are in fast exchange (relative to MRI acquisition times) with hydrogen atoms on water molecules, which account for more than 90% of all hydrogen atoms. As a result, the acquired T_2_ value in a typical MRI experiment is significantly reduced by the created polymer.

### 4.1. Radiation Chemistry

The water content in polymer gel dosimeters is generally in the order of 90%. Similar to Fricke gel dosimeters, the radiation-induced dissociation of water molecules results in highly reactive radicals and ions (Figure 1). These radiolytic water products (Table 1) initiate the polymerization reaction of the monomers. The decomposition of reactive intermediates in the chemical stage (after 1 µs) can be summarized as a simplified reaction of which the reaction rate is proportional to the absorbed dose.

(14) $$H_{2}O\overset{{\;k}_{D\;}}{\to }2R^{•}$$

where $R^{•}$ are the primary radicals, and $k_{D}$ is the radiation dose-dependent decomposition rate. From the six primary radiolytic products, the three radicals that are most involved in the initiation of monomers are: $e_{\mathrm{aq}}^{-}$, ${\mathrm{OH}}^{•}$, $H^{•}$. The rate of radical production R_D_ in units of mol/s is given by:

(15) $$R_{D}\left(R^{•}\right)=\;\left(G_{e_{\mathrm{aq}}^{-}}+G_{{\mathrm{OH}}^{•}}+G_{H^{•}}\right){\;\dot{D}\;m}_{w}$$

where $G_{i}$ is the chemical yield of primary radicals ($i={\;e}_{\mathrm{aq}}^{-}$, ${\mathrm{OH}}^{•}$, $H^{•}$) in units of mol/J, $\dot{D}$ is the dose rate expressed in Gy/s and $m_{w}$ is the mass of the irradiated water. The chemical yields of the three radicals produced by X-rays and gamma-rays in liquid water are, respectively, [103]: 2.8 × 10^−7^ mol/J, 2.9 × 10 ^−7^ mol/J and 0.57 × 10^−7^ mol/J.

These primary radicals initiate the polymerization of monomers or polymers containing a double bond by binding with an electron of the double bound.

(16) $$R^{•}+M_{n}\overset{k_{I}\left(n\right)}{\to }{\mathrm{RM}}_{n}^{•}$$

The polymerization reaction kinetics at the short time scale can be studied by using pulse radiolysis [39,209]. For acrylamide (AAm), one of the most commonly used monomers in polymer gel dosimeters, the three initiation reactions become:

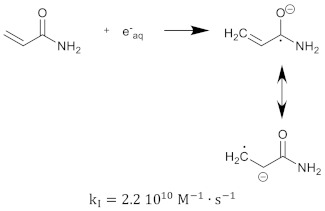
(17)

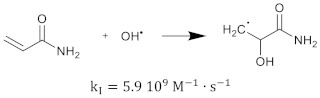
(18)

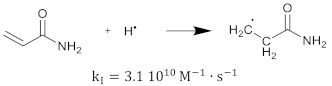
(19)
where $k_{I}$ is the reaction rate constant for each of the initiation reactions. In the reaction with the aquatic electron, various canonical forms of the electron adduct coexist.

The hydrated electron reacts with the monomers by the formation of a radical anion that can be further neutralized by a proton at low pH [210,211]. Similar initiation reactions occur for the cross-linker. The most used cross-linker in polymer gel dosimeters is N,N’-methylene-bis-acrylamide (Bis) [212,213,214].

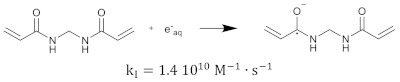
(20)

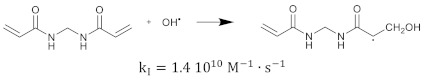
(21)

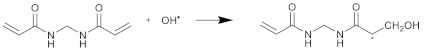
(22)

After some polymer is created, some of the polymer structures contain double bonds as a result of the cross-linker. These polymer structures can again react with primary radicals. The initiation reaction rate constant $k_{I}$ depends on the size of the polymers (i.e., the number of repetitive monomer units). It can be expected that the reaction rate will be smaller for larger polymers as the reactions are diffusion controlled [215], and reactive sites on larger polymer structures will be shielded [216,217]. This implies that the reaction rate $k_{I}$ can be seen as a function of the number of monomer units n [218]. Note that on the molecular level, it is not only the size of the polymer that is determining the reaction rate but also the shape of the molecule and the location of the reactive groups (double bonds) on the polymer. However, on a macroscopic scale, one may think of a statistical average of the different configurations of co-polymers. The created monomer radicals can further react with fresh monomers, which then form another polymer radical that can further react with new monomers or already formed polymer chains. As the cross-linking monomers have two double bonds on the same molecule, there can be pendant double bonds in the cross-linking polymer. This propagation reaction can be written in the generalized form:

(23) $${\mathrm{RM}}_{n,d1}^{•}+M_{m,d2}\overset{k_{p}\left(n,m,d2\right)}{\to }{\mathrm{RM}}_{\left(n+m\right),\left(d1+d2-1\right)}^{•}$$

where ${\mathrm{RM}}_{n,d1}^{•}$ is a polymer radical consisting of n monomer units and d1 available double bonds. $M_{m,d2}$ is a polymer chain with m monomer units and d2 pendant double bonds. In general, the reaction rate $k_{p}$ depends on the number of monomer units on both reacting molecules and on the number of pendant double bonds on the reacting polymer d2. Initially, there are no polymers in the gel and both n and m are equal to one, d1 is then zero and d2 is either 1 or 2 depending on whether it is a linear monomer or cross-linking monomer. In practice, for simplicity, a single reaction rate constant $k_{p}$ is considered.

The cross-linking monomer radical can also exhibit cyclization [35], as shown in Equation (24).

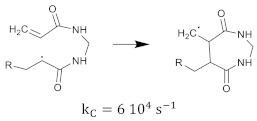
(24)

Termination of the polymerization reaction takes place by the combination of two radicals or by disproportionation. The growing polymer-radical may also terminate by transfer of the radical group to other molecules.

(25) $${\mathrm{RM}}_{n,d1}^{•}+{R\prime M}_{m,d2}^{•}\overset{k_{t}\left(n,m\right)}{\to }{\mathrm{RM}}_{\left(n+m\right),\left(d1+d2\right)}\;R\prime \;\;\;\left(n\leq m\right)$$

Typical chain transfer constants $C_{M}=k_{\mathrm{trans}}/k_{p}$ of radicals are in the order of 10^−3^ to 10^−4^ [219]. Chain transfer may occur with the growing polymer but also with the gelatine biopolymer. The decrease in the polymerization rate with increasing gelatine concentration provides some evidence of gelatine moderating the polymerization, possibly through chain transfer reactions or through scavenging of initiating fragments by the gelatine molecules [31].

In the most ideal case of a linear monomer in solution and considering only decomposition, initiation, propagation and termination, the polymerization rate expressed in terms of monomer bound in the form of a polymer can be written as:

(26) $$R_{p}=k_{p}\left[M^{•}\right]\left[M\right]=k_{p}\sqrt{\frac{R_{D}\left(R^{•}\right)}{k_{t}}}\left[M\right]$$

The ratio of reaction rate constants at 25 °C for AAm [220] in aqueous solution is $k_{p}/\sqrt{k_{t}}=4.7{\;M}^{-1/2}s^{-1/2}$ and for Bis [196] in aqueous solution $k_{p}/\sqrt{k_{t}}=\;9.85{\;M}^{-1/2}s^{-1/2}$. For a total comonomer concentration of 6% (*w*/*w*) (6%T) and a fraction of 50% cross-linker (50%C), the polymerization rate at a standard dose rate of 4 Gy/min becomes $R_{p}=0.392$ mM/s for AAm and $R_{p}=0.406$ mM/s for Bis. Assuming first order kinetics, a radiation dose of 10 Gy would result in a polymerization of 13.4% AAm and 26% Bis. A radiation dose of 48 Gy is needed to polymerize half of the Aam, and a radiation dose of 23 Gy is needed to polymerize half of the Bis. Experimentally, a two-fold ratio in Bis consumption and AAm consumption was found in AAm/Bis polymer gel dosimeters experimentally using FT-Raman spectroscopy [29,30]. However, the FT-Raman measured half-dose values of the exponential consumption of AAm and Bis were 8.36 Gy and 4.52 Gy, respectively. Several mechanisms may be responsible for this discrepancy. One of the mechanisms that could explain a higher polymerization rate in a polymer gel as compared to the polymerization rate in an aqueous solution is the difference in viscosity.

In the presence of oxygen during irradiation, peroxide radicals are formed that quickly terminate the polymerization chain reaction.

(27) $$R^{•}+O_{2}\overset{k_{O1}}{\to }{\mathrm{ROO}}^{•}$$

(28) $${\mathrm{RM}}_{n}^{•}+O_{2}\overset{k_{O2}\left(n\right)}{\to }{\mathrm{RM}}_{n}{\mathrm{OO}}^{•}$$

Recombination of the peroxides with growing polymer radical chains occurs according to:

(29) $${R\prime \mathrm{OO}}^{•}+R^{•}\overset{k_{R1}}{\to }R\prime \mathrm{OOR}$$

(30) $${\mathrm{ROO}}^{•}+{R\prime M}_{n}^{•}\overset{k_{R2}\left(n\right)}{\to }{\mathrm{ROOR}\prime M}_{n}$$

(31) $${R\prime M}_{n}{\mathrm{OO}}^{•}+R^{•}\overset{k_{R3}\left(n\right)}{\to }{R\prime M}_{n}\mathrm{OOR}$$

(32) $${R\prime M}_{n}{\mathrm{OO}}^{•}+{\mathrm{RM}}_{m}^{•}\overset{k_{R4}\left(n,m\right)}{\to }{R\prime M}_{n}{\mathrm{OO}\prime M}_{m}R$$

An oxygen concentration dependent inhibition in the R_2_-dose response was clearly observed in the low dose region of polymer gel dosimeters [221]. Oxygen can be removed from the gel by purging the gel solution with inert gasses such as nitrogen or argon gas or by use of an antioxidant [36,222]. Fuxman et al. [35,223] developed a numerical reaction kinetics model for polyacrylamide gel dosimeters. In this reaction–diffusion model, the reaction scheme is extended with transfer reactions to monomer and initiation with gelatine radicals. Both an aqueous phase and a polymer phase are considered with separate reaction rate constants for each of the phases. The effect of different dose rates, different monomers and gelatine concentrations on the polymerization rate and edge enhancement effects at the boundary of a radiation field was demonstrated.

At high conversions of monomers, an increase in viscosity hinders termination by the mutual interaction of growing chains but has less effect on the propagation reaction (Equation (23)), because diffusion of the small monomer molecules is less affected by the increased viscosity. As a result, the rate of polymerization shows an increase with high conversion of monomers [224]. This manifestation of auto-acceleration is also called the Trommsdorff effect [223]. It was reported that in systems in which the polymer precipitates from the solution by the creation of a heterogeneous gel system, the increase in viscosity takes place very rapidly even at low conversions [225]. This effect was illustrated through mathematical models of dispersion radical polymerization kinetics [218]. The increasing size of polymer aggregates in polymer gel dosimeters as observed by optical turbidity spectra [226] was also attributed to the Trommsdorff effect. It is not completely clear yet if the non-linear response in the low-dose region (seen from 0 to 1 Gy) of several gel systems [50] also reflects this sudden change, or if the non-linearity is due to chemical reactions with other molecular species in the gel. Another source of non-linearity of the radiation dose response is attributed to a different reaction rate of the two comonomers. The different reaction rates of the co-monomers lead to a shift in the instantaneous relative co-monomer concentration [227,228]. Baselga et al. distinguish three different reaction steps in the cross-linking co-polymerization of an AAm/Bis aqueous solution: a pre-gel step, gelation and post-gel reactions [229]. In the pre-gel step, the cross-linked polymer particles are richer in Bis. At the gel point, the rate of reaction increases for both co-monomers, but the increase is larger for AAm. During gelation, the pre-gel particles are joined by chains that are slightly richer in AAm. The post-gel phase is characterized by slow cross-linking as a result of shielding of the radical group by the co-polymer chains [28,217] and reorganization of the polymer networks [31]. In many publications, this non-linear relation in the low-dose region is often ignored, and a mono-exponential saturation curve or linear fit is applied to the dose-R_2_ plots of polymer gel dosimeters. In practice, the dose–R_2_ response curve of polyacrylamide gel dosimeters can be very well fitted with a bi-exponential function. The different reaction rates of AAm and Bis are also translated in a different dose sensitivity between gels with different cross-linker fractions [29]. The dose sensitivity for PAG gel dosimeters is maximum for equal weight fractions of monomer (AAm) and cross-linker (Bis) (i.e., for 50%C (*w*/*w*)) [50].

**Figure 3:**
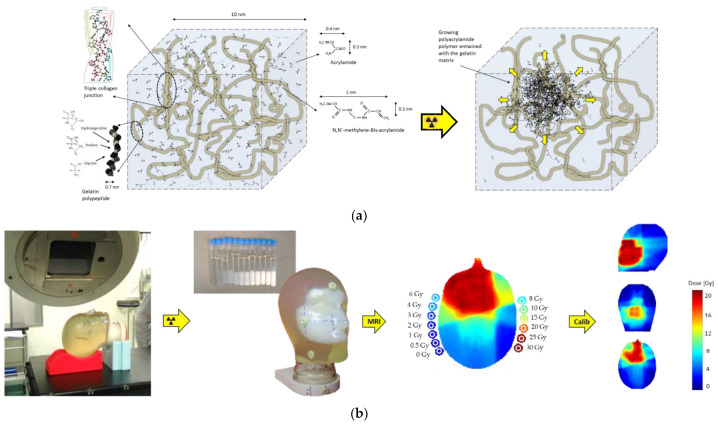
Schematic representation of the polymer gel before and after irradiation (**a**). Upon radiation, an interpenetrating polymer network is created that is entangled with the gel matrix. Head phantom and calibration vials filled with polymer gel demonstrate a visible change in opacity upon irradiation (**b**). R_2_ maps can be calibrated to dose by use of the R_2_ values measured in the calibration vials (**b**).

The PAG polymer gel dosimeter is believed to consist of an interpenetrating network of highly cross-linked polyacrylamide aggregates with a gelatine hydrogel matrix (Figure 3). In acrylic acid-based gel dosimeters, the growing acrylic acid copolymer is believed to graft onto the gelatine matrix [67,230]. Acrylic acid or methacrylic acid radicals may bind with hydroxyl- or amino-groups on the gelatine polypeptide chain, on which more monomer units may propagate until another gelatine chain is encountered.

It is believed that as a result of the change in relative monomer/cross-linker ratio during the radiation and the varying size of polymer aggregates, the intertwined polyacrylamide network is not uniform on the microscopic level. Other studies on PAG gels have also shown large heterogeneities with different gel densities [32,33,228,231].

The polymerization reaction in polymer gel dosimeters is found to be exothermal, which can be measured using a thermocouple [33] or a fluoroptic thermometry system [23,232]. The exothermal reaction leads to an increase in temperature, which in itself also influences the reaction kinetics [223]. Because the radiation exposure varies from point-to-point in a typical radiotherapy treatment, some uncertainty in the dose distribution can be expected. For PAG gel dosimeters it has been shown that the exothermal reaction in a typical dosimetry experiment can result in a dose uncertainty of 4% [232].

### 4.2. MRI Contrast Mechanism and Dose Sensitivity

The transverse relaxation in polymer gel dosimeters can be described by a model consisting of three components: a mobile water pool, a growing polymer network and a gel matrix. If the protons are in fast exchange, the observed relaxation rate can be described as a first order equation by a weighted average of the relaxation rates of the three proton pools:

(33) $$R_{2}=f_{\mathrm{mob}}^{H}R_{2,\mathrm{mob}}+f_{\mathrm{pol}}^{H}R_{2,\mathrm{pol}}+f_{\mathrm{gel}}^{H}R_{2,\mathrm{gel}}$$

where $f_{\mathrm{mob}}^{H}$, $f_{\mathrm{pol}}^{H}$ and $f_{\mathrm{gel}}^{H}$ are the molar fractions of the mobile water, polymer network and gel matrix agent hydrogen pools, respectively, and $R_{2,\mathrm{mob}}$, $R_{2,\mathrm{pol}}$ and $R_{2,\mathrm{gel}}$ are the apparent relaxation rates of the corresponding pools. The apparent relaxation rates consist of the intrinsic relaxation rate of each proton carrying chemical compound and a contribution of magnetization transfer with other proton pools. The intrinsic relaxation rates are determined by the molecular mobility in accordance with the Bloembergen-Pound-Purcell (BPP) theory [107], as further extended by Woessner [233]. The decreased molecular tumbling of protons on the polymer results in a large increase in transverse relaxation (shorter T_2_), while the longitudinal relaxation is not much affected. In PAG gel dosimeters, the gelatine hydrogen proton pool is assumed to be barely affected by the polymerization.

**Figure 4:**
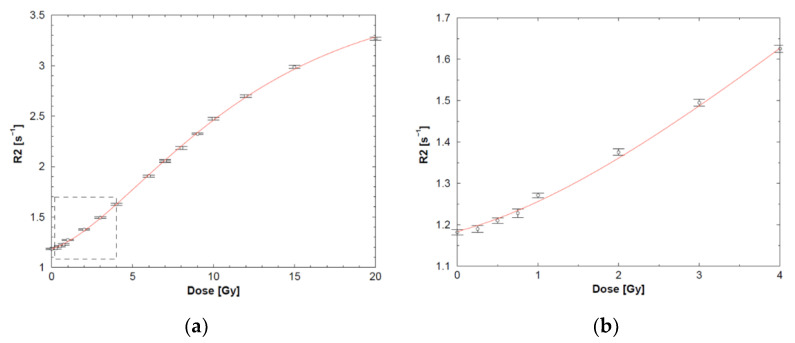
Typical sigmoidal course of the dose-R2 plot of an anoxic PAG gel dosimeter (**a**) with a magnified view of the low-dose region (**b**) demonstrating a non-linear increase in R_2_ with the absorbed dose. Adapted from [28] with permission from the Institute of Physics and Engineering in Medicine, Copyright 2000 IOP Publishing.

The R_2_-dose response follows typically a sigmoidal response (Figure 4).

The half-value dose D_1/2_ (i.e., the dose for which the change in R_2_ reaches half of its saturation value), R_2_-dose sensitivity and dynamic R_2_-range of the different polymer gels are listed in Table 3. Note that these are only indicative values as some variations are seen depending on measurement temperature and antioxidant used.

**Figure 5:**
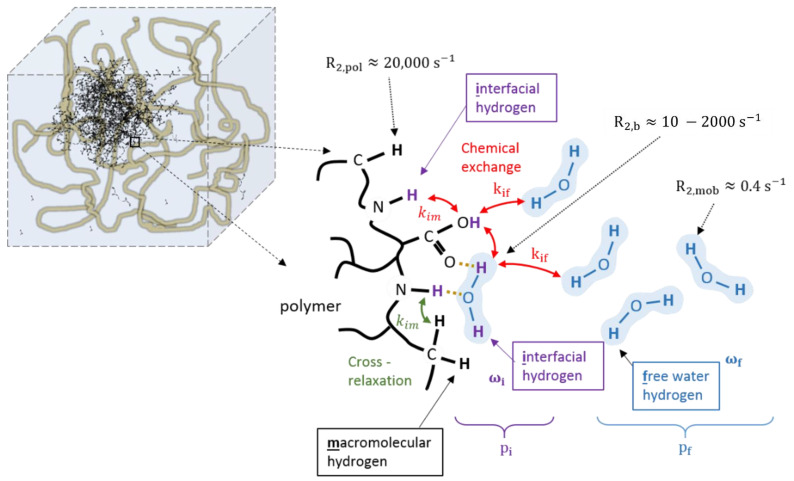
Different hydrogen proton pools near the polymer backbone (black): macromolecular non-exchangeable hydrogen pool (black), exchangeable interfacial hydrogen on either the polymer or irrotational bound water (purple) and free water hydrogen pool (blue). Each group of hydrogen protons has an R_2_ relaxation rate depending on their molecular mobility as indicated. Chemical exchange occurs between exchangeable interfacial hydrogen protons and free water hydrogen protons (k_if_). Cross-relaxation occurs between interfacial hydrogens and macromolecular polymer hydrogens (k_im_).

The big differences in dose response for different monomers cannot be explained on the basis of the BPP theory only. It was shown that the relaxation rates are significantly affected by the exchange rate of magnetization [237,238,239] between the different proton pools, either through cross-relaxation or chemical exchange. Figure 5 provides a more sophisticated model of the interactions between hydrogen proton pools near the polymer. In this model, a distinction is made between exchangeable (interfacial) hydrogen protons and non-exchangeable macromolecular hydrogen protons. Hydrogen protons of water molecules that are irrotationally bound onto the polymer surface can be considered part of the interfacial proton pool. Chemical exchange may occur between interfacial hydrogen protons and free water protons with a rate constant k_if_. Cross-relaxation may still occur between interfacial hydrogen protons and non-exchangeable hydrogen polymer protons. Cross-relaxation only plays a role in the longitudinal relaxation.

For the transverse relaxation, the chemical exchange between the interfacial and free hydrogen pool creates an additional contribution if there exists a difference in resonance frequency between the two proton pools [22].

(34) $$R_{2}=f_{f}^{H}R_{2f}+f_{i}^{H}R_{2i}+f_{f}^{H}f_{i}^{H}\left(\frac{\tau_{f}\tau_{i}}{\tau_{f}+\tau_{i}}\right){\left(\omega_{f}-\omega_{i}\right)}^{2}$$

Here, $f_{f}^{H}$ and $f_{i}^{H}$ are the fraction of free water protons and intermediate exchangeable macro-molecular protons, respectively, ($f_{i}^{H}+f_{f}^{H}=1$), $\tau_{f}=1/k_{\mathrm{fi}}$ and $\tau_{i}=1/k_{\mathrm{if}}$ are the lifetime in each of the pools and $\omega_{f}$ and $\omega_{i}$ are Larmor frequencies in each of the pools.

### 4.3. Radiation Properties

While the precision of the dose reading in gel dosimeters is determined by the dose sensitivity and the readout method, the accuracy of the radiation dosimeter depends on dosimeter-specific radiation properties [23,24,25]. The radiation properties of different polymer gel dosimeters have been studied by several research groups and are summarized in Table 4. Table 4 illustrates that not all radiation properties have been studied for all polymer gel formulations.

#### 4.3.1. Stability

**Figure 6:**
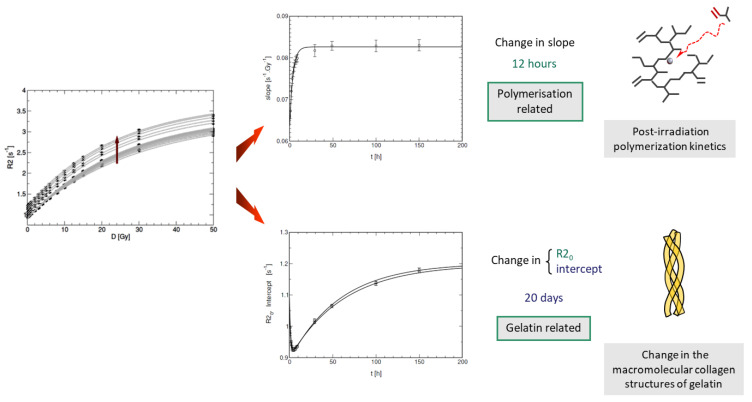
Two kinds of instability of the dose-R_2_ response of polyacrylamide-based gel dosimeters can be considered [28]. One kind of instability, which occurs on a timescale of 12 h, is responsible for a change in the dose-R_2_ sensitivity and is related to the post-irradiation polymerization kinetics. Another kind of instability affects the R_2_ offset after manufacturing, occurs over a time span of several weeks and is related to the gelation and ‘ageing’ of the gelatine biopolymer. Adapted from [43] with permission from the Institute of Physics and Engineering in Medicine, Copyright 2000 IOP Publishing.

It was found that the R_2_ value of unirradiated gelatin-based polymer gel dosimeters increased slightly from the moment of fabrication till several days after (Figure 6). To describe this long-term instability of polymer gel dosimeters, the gelatine water pool is divided in two separate hydrogen pools consisting of structured water and non-structured bound water [28]. The structured water in gelatine can be attributed to water bound to the polypeptide chains that stabilize the triple helices or aggregates of tropocollagen [257], which was confirmed by measurements of optical activity of the gel over time [28].

In methacrylic acid-based gel dosimeters such as MAGAT, the post-irradiation polymerization occured in the first 2 min after radiation exposure [93], more than two orders of magnitude faster than what was found in polyacrylamide gel dosimeters and was first observed on an MR-Linac. This faster reaction rate is attributed to the different polymerization kinetics (i.e., grafted polymer on gelatine versus heavily cross-linked polymer aggregates). A similar temporal response was found optically, but a larger lag-phase in the order of 1.5 min was found [258], whereas the change in R_2_ was nearly immediate upon exposure. This may be attributed to the critical size of polymer structures that needed to be formed in the gel before any significant light-scattering occured, while in NMR, the change in molecular mobility was a more rapid physical change that affected the R_2_. To decrease the retardation in the dose registration of gel dosimeters is an ongoing challenge for inline real-time radiation dosimetry on MR-Linacs.

#### 4.3.2. Spatial Integrity

**Figure 7:**
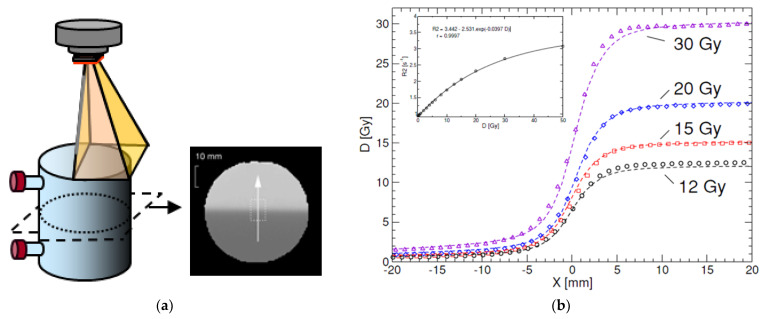
Radiation of a PAGAT gel phantom with a half-blocked field to create a sharp penumbra (**a**). While the registered dose up to 20 Gy matches with the expected dose distribution, as measured by use of a diamond detector, an overshoot in dose can be seen for 30 Gy (**b**). The measured dose profiles are acquired 10 h post-radiation. The dose penumbra normalizes after 6 days post-radiation [43]. Adapted from [43] with permission from Institute of Physics and Engineering in Medicine, Copyright 2000 IOP Publishing.

A related physico-chemical mechanism that can have a significant effect on the accuracy of dose registration is the possibility of an overestimation of the dose near steep dose gradients [43]. This phenomenon is attributed to the diffusion of unreacted monomers into regions with long-living polymer radicals after radiation exposure [36,47]. In PAGAT gel dosimeters (Figure 7), the dose overestimation was visible for a dose of 30 Gy at 10 h after radiation exposure. This overestimation disappeared after several days as more monomers diffused towards the high dose region and reacted with long-living polymer radicals. This renormalization was not observed in anoxic PAG gel dosimeters or in normoxic MAGAT gel dosimeters [43,45,47]. These differences between the different gel dosimeter types may be attributed to the lifetime of the polymer radicals. The occurrence of these overshoots was simulated by the use of simplified analytical models [47] and more elaborate chemical reaction modeling [36]. For the MAGAT gel dosimeters and anoxic PAG gel dosimeter, the overshoots were also more pronounced. The difference in temporal integrity between different gel dosimeters illustrates the importance of an in-depth characterization of the radiation properties of new polymer gel formulations before clinical usage.

#### 4.3.3. Dose-Rate Dependent Dose-R_2_ Response

From the complexity of diffusion-controlled reaction mechanisms discussed in Section 4.1, it is not surprising that the dose response is dependent on the rate of radiation-induced radical production and, thus, on the dose rate. In a typical dose distribution, both the absorbed dose and dose rate vary from voxel to voxel. It is, therefore, required that the dependence of the dose response on the dose rate is as low as possible. The dose-rate dependency is largely dependent on the kind of monomers in the polymer gel dosimeter. It is also expected that the gel matrix has some influence on the dose-rate dependence as it influences the diffusion of polymerization products. PAGAT and VIPAR gel dosimeters are less dose-rate dependent than MAGIC and MAGAT gel dosimeters [43,49]. Remarkably, higher concentrations of antioxidants in the MAGIC gel result in a lower relative dose-rate dependence but at the cost of a lower dose sensitivity [49].

#### 4.3.4. Oxygen Contamination

In contrast to the first polymer gel dosimeters that were fabricated and stored under anoxic atmospheric conditions, ‘normoxic’ gel dosimeters were constructed on the lab bench without the need for an expensive and complicated laboratory set-up. However, it is a mistake to assume that normoxic gel dosimeters are completely insensitive to oxygen. Normoxic gel dosimeters still require casting materials that avoid infiltration of ‘fresh’ oxygen [259,260]. Large amounts of oxygen in the gel dosimeter results in inhibition of the polymerization reaction, while small amounts result in a promotion of polymerization. It was also found that a large amount of the antioxidant results in a decrease in the radiation sensitivity [260]. To guarantee the homogeneous distribution of oxygen and the antioxidant, it is strongly advisable to use impermeable cast materials such as Barex™ or glass and to make sure that the antioxidant is well mixed in the final gel phantom [24].

### 4.4. Applications of Polymer Gel Dosimeters

Polymer gel dosimetry has been applied in a large variety of radiation experiments and clinical 3D dose verifications. A polymer gel dosimetry experiment involves many steps from gel fabrication to readout and data analysis. In order to obtain reliable dose measurements, a rigorous approach needs to be followed. A typical gel dosimetry experiment easily takes 2–3 days to perform, which depends also on the availability of clinical instrumentation such as MRI scan time. Until now, polymer gel dosimetry has not been considered as a routine dosimeter to check every radiotherapy patient treatment but is rather used to provide an end-to-end dosimetry test of a class solution of treatments [24,261,262]. An overview of applications until 2008 is also given in the topical review paper on polymer gel dosimetry [21]. We hereby restrict to a few examples.

#### 4.4.1. Intensity Modulated Treatments

**Figure 8:**
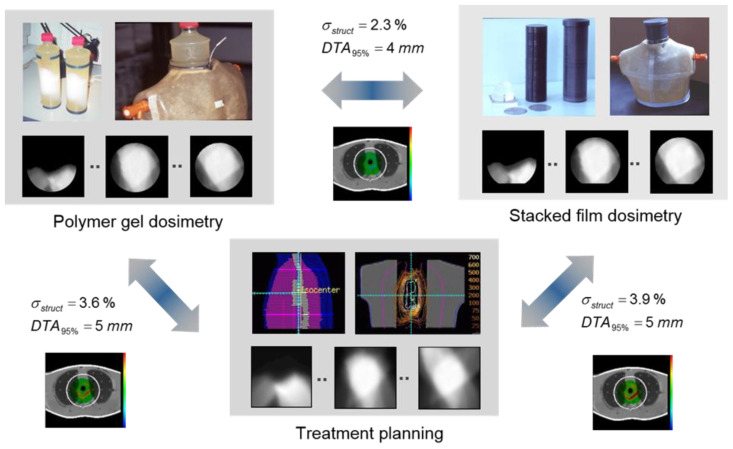
IMRT treatment of a mediastinal tumor consisting of 6 non-coplanar beams sparing the spinal cord. Comparison with stacked radiographic film dosimetry and treatment planning revealed a good correspondence, which resulted in confidence about the whole treatment chain. The dosimeter gel was an anoxic polyacrylamide gelatine (PAG) gel that was cast in a cylindrical glass bottle and was slid into a cylindrical cavity of a thoracic phantom.

Polymer gel dosimetry for a non-coplanar IMRT treatment of a mediastinal tumor [263] is shown in Figure 8. In this experiment, a thorax phantom was constructed that had a cylindrical cavity that was able to accommodate a gel insert and an insert with a stack of 20 circular radiographic films for comparison. The dose information obtained with polymer gel dosimetry was compared with the dose distribution measured by the stack of radiographic films. Quantitative comparison was made on the basis of pixel-wise root-mean-square deviations and a distance-to-agreement (DTA) metric. The effect of photon beam energy on the treatment was also measured. In a later experiment, a similar thorax phantom including a lung cavity that could be filled with water or air was used to study the effect of air cavities on the dose distribution [264].

**Figure 9:**
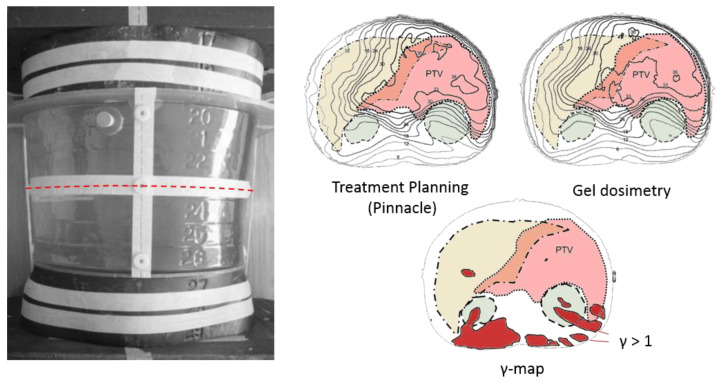
Whole abdominopelvic IMAT palliative treatment of patients with relapsed ovarian cancer [258]. The abdominopelvic gel dosimeter phantom consists of a vacuum molded Barex™ cast filled with a normoxic PAGAT gel and was surrounded by slaps of the Rando^®^ phantom. The yellow shaded area corresponds to the liver, the green shaded areas to the kidneys and the pink shaded area corresponds to the planning target volume (PTV). The red regions in the gamma map are regions where gamma exceeds 1. Adapted from [265] with permission from the Institute of Physics and Engineering in Medicine, Copyright 2000 IOP Publishing.

An example of an intensity modulated arc treatment (IMAT) on a 10 liter anthropomorphic Barex™ phantom filled with PAGAT gel is shown in Figure 9. A whole abdominopelvic IMAT of a relapsed ovarian cancer was validated in this study [265]. To obtain reliable dose maps in such a large phantom, compensation for B_1_ field heterogeneity was required and temperature drift as a result of absorbed radiofrequency RF energy was compensated for on the pulse sequence level [72]. Comparisons of 3D dose distributions were made by using a single metric that comprised both dose differences and the distance-to-agreement [265,266,267]. This metric is referred to as the gamma-index (not to be confused with the extension of the factorial gamma-function). The gamma-index γ in a reference point ${\vec{r}}_{r}\left(x_{r},y_{r},z_{r}\right)$ is defined as the minimum of the 4D Euclidian metric Γ taken over a set of all neighboring evaluation points with coordinates ${\vec{r}}_{e}\left(x_{e},y_{e},z_{e}\right)$:

(35) $$\gamma \left({\vec{r}}_{r}\right)=\underset{\left\{{\vec{r}}_{e}\right\}}{\min}\left\{\Gamma \left({\vec{r}}_{r},{\vec{r}}_{e}\right)\right\}$$

where the 4-D Euclidian metric is given by:

(36) $$\Gamma \left({\vec{r}}_{r},{\vec{r}}_{e}\right)=\sqrt{\frac{{\Delta r}^{2}}{{\delta r}^{2}}+\frac{{\left(D\left({\vec{r}}_{r}\right)-D\left({\vec{r}}_{e}\right)\right)}^{2}}{{\delta D}^{2}}}$$

where Δr is the Euclidean distance between the reference point and any evaluation point ($\Delta r=\sqrt{{\left(x_{r}-x_{e}\right)}^{2}+{\left(y_{r}-y_{e}\right)}^{2}+{\left(z_{r}-z_{e}\right)}^{2}}$), $D\left({\vec{r}}_{r}\right)$ and $D\left({\vec{r}}_{e}\right)$ are the dose values in the reference dose map and the evaluation dose map, respectively, and δr and δD are evaluation criteria for the ‘allowable’ distance and dose, respectively.

In some publications, the gamma pass rate, the percentage of points for which γ < 1, is used as a global evaluation criterium of two dose distributions. It is important that this value is used with great care as the gamma pass rate depends on the region in which gamma is evaluated. Moreover, not all points in a dose distribution may be equally important. Indeed, low-dose regions at a large distance from the PTV may not be as important as regions between the PTV and an organ-at-risk (OAR).

Several other studies have demonstrated the use of polymer gel dosimetry for the validation of intensity modulated treatments in either canonical phantoms [268,269,270,271,272,273,274,275,276,277,278,279] or anthropomorphic shaped phantoms [72,163,280,281,282,283].

#### 4.4.2. Stereotactic Radiosurgery and Gamma Knife

As most polymer gel dosimeters are used with larger doses than those provided in a single fraction, many studies have focused on stereotactic radiosurgery where larger doses are delivered [73,83,234,284,285,286,287,288,289,290,291,292,293,294,295]. In most of the studies, the emphasis of the treatment validation experiment is on the spatial accuracy rather than on the absolute dose delivery.

#### 4.4.3. Brachytherapy

Polymer gel dosimeters exhibit an R_2_-dose response that is measurable up to two orders of magnitude, which makes them potentially suitable for the evaluation of brachytherapy dose validation studies. However, care is required for brachytherapy of single point sources, as a loss of spatial integrity was found in regions with very steep dose gradients and that are close to the monomer depletion zone [67]. This phenomenon was also studied by mathematical chemical kinetics modeling [296]. The steep dose gradients near a brachy source, in particular point sources, also impose high demands on the imaging resolution as partial volume effects lead to an underestimation of dose [67,297,298]. Additional care is required to avoid image distortions caused by magnetic susceptibility related field heterogeneity around the catheter [67] and contamination of the gel by oxygen from the catheter [67,299]. Polymer gel dosimetry was applied for treatment verification around intravascular sources [299,300,301,302,303], intracavitary brachytherapy [242,304,305,306,307,308] and interstitial brachytherapy [309,310]. As with external radiotherapy, polymer gel dosimetry was found of particular importance in end-to-end delivery quality assurance [311,312].

#### 4.4.4. Proton and Ion Therapy

Several attempts were undertaken to map the dose distribution in proton [235,313,314,315,316,317,318,319,320] and ion therapy [321,322]. Unfortunately, in most studies, ‘quenching’ of the measured dose in the Bragg peak occurred, leading to an underestimation of the dose in the Bragg peak. The quenching is quantitatively expressed in terms of a relative effectiveness (RE) or efficiency, which is defined as the ratio of radiosensitivity between high LET radiation (protons or heavy ions) and low LET radiation (i.e., photons) [34,313]. The relative effectiveness is found to be dependent on the gel type and the type of the radiation. The RE increases with increasing particle energies, which corresponds to lower LET. It was also noted that partial volume effects as a result of limited imaging resolution may cause an additional decrease in the relative effectiveness [322]. High resolution imaging at higher magnetic field strengths is, therefore, recommended [323]. Another study applies optical laser scanning to read out the gel dosimeter [320], but great care is required in the interpretation of more complex dose distributions as light scattering can severely compromise the accuracy.

#### 4.4.5. Boron Neutron Capture Therapy

In boron neutron capture therapy (BNCT), the patient is injected with a tumor-localizing boron-10 isotope that has the capacity to capture low energetic neutrons upon which alpha particles and gamma radiation are released. Dosimetry in BNCT is not straightforward because of the different radiation contributions. Polymer gels doped with boracic acid were irradiated with epithermal neutrons and read out with quantitative MRI [324,325]. In addition, non-doped polymer gel dosimeters were applied to study the dose in epithermal neutron beams without boron [326]. Theoretically, on the basis of stochiometric calculations, it is concluded that the response of PAG gel dosimeters is correlated with the radiation transport in BNCT similar to brain tissue [327], but more convincing proof is needed to correlate the gel measured dose with the actual absorbed dose.

#### 4.4.6. Dosimetry near Non-Water-Equivalent Tissues

Polymer gel dosimeters are tissue equivalent in terms of electron density and stopping power [56] for most body regions such as the head, neck, abdomen and limbs. However, dose registration in body regions with low-density, such as the lungs, or high-density, such as bone, is more challenging. Lung tissue has a lower electron density than soft tissue. The difference in density results in a difference in radiation interactions and the absorbed dose. The composition of polymer gel dosimeters can be modified to make them lung equivalent. A lower electron density can be obtained by beating the gel into a hydrogel foam [328] or by adding low density Styrofoam beads [329]. While the dose in the lung-equivalent gel dosimeter can be derived from quantitative MRI maps of magnetization transfer or R_2_ maps, the electron density can be derived from proton density maps [328]. The dose distribution of radiation beams passing through non-water equivalent structures including bone structures and air was also studied [264,330].

#### 4.4.7. Diagnostic Radiation Dosimetry

Polymer gel dosimetry was applied to determine the dose distribution in computer tomography (CT) [331]. A routine CT quality assurance parameter that can be extracted from the gel measured dose maps is the computer tomography dose index (CTDI). For diagnostic radiation dosimetry, a high-sensitivity gel dosimeter is required, and thus, a methacrylic acid-based gel dosimeter is used for these studies. In another study, the dose sensitivity was enhanced by adding iodine as a radiosensitizer to a NIPAM gel [332]. Remarkably, where at higher dose rates the dose sensitivity decreases with increasing dose rate, at lower dose rates (5–8 cGy^−1^ min^−1^) as encountered in diagnostic radiation, the dose sensitivity is found to increase with increasing dose rate [333]. This is attributed to the lack of radical recombination at these low dose rates. The effect of radiation from CT scanning on polymer gel dosimeters was also studied in the light of X-ray scanning of gel dosimeters where the dose from X-ray CT could lead to an additional uncertainty if not accounted for [334].

#### 4.4.8. Radionuclide Dosimetry

Vials of normoxic polymer gel were doped with P-32 [335], I-131 [336,337] and Tc-99m [338], and the measured absorbed dose was compared with the Monte Carlo simulated dose. The dose sensitivity for internal radiation with P-32 [335] was approximately 40% higher than the dose response with an external 6 MV photon beam. This difference can be attributed to the large difference in dose rate between both forms of radiation.

## 5. Readout Systems

### 5.1. MRI Scanning

In theory, it is possible to use any MRI pulse sequence that generates images in which the signal intensity is uniquely correlated with the absorbed radiation dose (not necessarily linear). In theory, any conventional T_1_-weighted or T_2_-weighted sequence can be used. However, in practice, as a result of B_0_ and B_1_-field heterogeneity, the uniqueness between dose and signal intensity is not guaranteed with these T_1_- and/or T_2_-weighted sequences, which can severely compromise the accuracy. Quantitative T_1_ or T_2_ pulse sequences are obvious choices as the relaxation times are the major contrast parameters that are affected. The majority of the signal intensity artifacts related to B_0_- and B_1_-field magnetic field heterogeneity are absent in quantitative R_1_- and R_2_-maps. Historically, for Fricke gels, the focus has been on the longitudinal relaxation rate. While the R_2_-dose sensitivity is in the same order as the R_1_-dose sensitivity, the R_2_-offset of agarose-based Fricke gel dosimeters is relatively high, which results in a worse dose resolution, hence, the choice of quantitative T_1_ sequences above T_2_ sequences. However, for gelatin-based Fricke gel dosimeters, both T_1_ and T_2_ sequences can be applied. Because polymer gel dosimeters are not T_1_ sensitive, R_2_ mapping is preferred.

#### 5.1.1. R_1_ Mapping

Several MRI pulse sequences can be used to acquire R_1_ maps [338]. The most common pulse sequences are:

1. Spin echo (SE), gradient echo (GE) and rapid acquisition with relaxation enhancement (RARE) sequences: The repetitive sequence blocks in a SE sequence take the shape:
  (37) $${\left(90{}^{\circ}-\left[\frac{\mathrm{TE}}{2}\right]-180{}^{\circ}-\left[\frac{\mathrm{TE}}{2}\right]-\mathrm{SE}-\left[\mathrm{TR}-\mathrm{TE}\right]\right)}_{N_{\mathrm{ph}}}$$
  where the angles 90° and 180° correspond with the flip angles of the RF pulses, TE is the echo time and TR is the repetition time. N_ph_ is the number of phase encoding steps. In this group of sequences, different base images are acquired with varying TR. In the first approximation, where TE is significantly shorter than TR and for perfect 90° excitation and 180° refocusing pulses, the fitting signal equation becomes:
  (38) $$S_{f}={\;S}_{0}\left(1-e^{-R_{1}\mathrm{TR}}\right)e^{-R_{2}\mathrm{TE}}=S_{0}\prime \left(1-e^{-R_{1}\mathrm{TR}}\right)$$
  The parameter R_1_ for every imaging voxel can be obtained from Equation (38) by fitting each voxel on a voxel-by-voxel basis from a set of N base images acquired at various TR. For each voxel, the exponential term in R_2_TE is constant for fixed TE. It was shown that in order to fit relaxation rates, a $\chi^{2}$-minimization was preferable above least square fitting methods [74]. For a voxel with spatial coordinates (x, y, z), $\chi^{2}$ is given by:
  (39) $$\chi^{2}=\overset{N}{\underset{k=1}{\sum }}\frac{{\left(S_{\mathrm{image},\;k}-S_{f}\left(S_{0},R_{1},R_{2},\mathrm{TE},{\mathrm{TR}}_{k}\right)\right)}^{2}}{\sigma_{S}^{2}}$$
  where $S_{\mathrm{image},\;k}$ is the acquired signal intensity in the base image with index k acquired with echo time TE and repetition time ${\;\mathrm{TR}}_{k}$. The fit function $S_{f}\left(S_{0},R_{1},R_{2},\mathrm{TE},{\mathrm{TR}}_{k}\right)$ is given by the functional relation 38, where $S_{0}{\prime \;\mathrm{and}\;R}_{1}$ are the unknown fit variables. Correction factors and additional terms may be applied to compensate for imperfections in the RF pulses and when the value of TE is close to TR. It is also convenient to apply an image threshold filter to filter out background voxels. In addition to the parametric relaxation maps, parametric maps of fitting performance, such as Pearson correlation coefficients, can also be calculated.
2. *Saturation and inversion recovery sequences*: A second class of pulse sequences that can be employed to map R_1_ are the saturation and inversion recovery sequences. The *saturation recovery sequence* is of the form:
  (40) $$\left(90{}^{\circ}-G_{c}-\mathrm{TM}-90{}^{\circ}-\mathrm{GE}\right)$$
  Here, a 90° RF pulse is followed by a crusher gradient G_c_ that destroys the longitudinal and transverse magnetization. The longitudinal magnetization starts to recover after the 90° pulse. After a recovery time TM, the longitudinal magnetization component is turned into a measurable gradient echo (GE) signal by use of a second 90° pulse. The signal intensity in the saturation recovery sequence is given by:
  (41) $$S={\;S}_{0}\left(1-e^{-R_{1}\mathrm{TM}}\right)$$
  In an *inversion recovery sequence*, a 180° inversion pulse is applied.
  (42) $$\left(180{}^{\circ}-\mathrm{TI}-90{}^{\circ}-\mathrm{GE}\right)$$
  After an inversion recovery time TI, the longitudinal magnetization is turned into a measurable signal. The signal intensity acquired with the inversion recovery experiment is given by:
  (43) $$S={\;S}_{0}\left(1-\left(1-\cos\theta_{\mathrm{inv}}\right)e^{-R_{1}\mathrm{TI}}+e^{-R_{1}\mathrm{TR}}\right)$$
  where $\theta_{\mathrm{inv}}$ is the effective flip angle of the inversion pulse. In practice, the effective flip angle is considered a fitting variable to compensate for imperfect inversion over the entire slice. In the case of an inversion recovery experiment, it is important to acquire the real component of the signal instead of the magnitude of the signal. In principle, any kind of fast read out block can be applied after the 90° pulse, such as a spin echo, fast spin echo or a combination of a gradient echo train and spin echo train, also referred to as a GRASE readout. An additional attenuation of the signal in Equations (42) and (43) applies that is dependent on the readout sequence block but does not affect the longitudinal relaxation weighting factor. As a result, this additional attenuation can be absorbed in the factor $S_{0}$. For gel dosimetry, it is not recommended to use a fast echo planar imaging (EPI) readout because of the significant spatial distortions related to the low bandwidth in the phase encoding direction in combination with magnetic field inhomogeneity as a result of magnetic susceptibility differences between air and gel.
3. *Look-Locker sequences*: A fast sequence to acquire R_1_ maps is the Look-Locker sequence [321], of which the sequence building block takes the shape of Equation (44).
  (44) $$\left(180{}^{\circ}-{\left\{\tau -\alpha -\mathrm{GE}\right\}}_{n}\right)$$
  The Look-Locker sequence [339] is similar to the inversion recovery gradient echo sequence, but a faster readout is achieved by replacing the refocusing 90° pulse with a train of small flip angle pulses with flip angle α separated by a time τ. A small portion of the recovering longitudinal magnetization is flipped in the transverse plain by the α-pulses, which is read out as gradient echoes. Each acquired echo has a different T_1_ weighting and make up one k-space line in each base image. An R_1_-map is then reconstructed by fitting the signal intensity in the base images to a modified T_1_-relaxation function. A variation of the Look-Locker sequence is the TOMROP (T_1_ by multiple readout pulses) sequence. In this sequence the gradient echoes are grouped and interleaved with some recovery time to allow T_1_ relaxation to take place. Each group of gradient echoes is used as separate k-space lines in a T_1_-weighted base image. The different T_1_-weighted base images are then used to reconstruct an R_1_ map. The Look-Locker sequence is particularly sensitive to RF pulse imperfections. Additionally, changes in the flip angle distribution within the image (B_1_-field non-uniformity) affect the signal.
4. *Steady-state free precession sequences*: A sequence of RF pulse excitations separated by a time TR brings the NMR signal in a steady state. In between two successive pulses the frequency encoding gradients can be placed in such a way that two echoes are obtained.
  (45) $$\left(\alpha -\left[\frac{\mathrm{TE}}{2}\right]-\mathrm{GE}-\tau -\mathrm{SE}-\left[\frac{\mathrm{TE}}{2}\right]\right)$$
  Both the degree of T_1_ and T_2_ weighting are affected by the flip angle α. A special case of SSFP is when the net gradient area is zero at any of the three gradient axes during one TR interval. In this case (τ=0), only a single echo is acquired between each successive RF pulse. This pulse sequence is called ‘balanced SSFP’.

#### 5.1.2. R_2_ Mapping

While a standard spin echo sequence can be used to acquire base images with different echo times, a faster imaging sequence is the *multi-spin-echo (MSE)* sequence. The MSE sequence is similar to the RARE sequence discussed above as it consists of a 90° excitation pulse followed by a train of 180° pulses but differs in that every spin echo signal is stored in a different k-space matrix, resulting in a set of N_echo_ different T_2_-weighted images. A typical multi-spin-echo pulse sequence scheme is the Carr–Purcell Meiboom–Gill (CPMG) sequence is shown in Equation (46).

(46) $${\left(90_{x}{}^{\circ}-{\left\{\left[\frac{\mathrm{TE}}{2}\right]-180_{y}{}^{\circ}-\left[\frac{\mathrm{TE}}{2}\right]-\mathrm{SE}\right\}}_{N_{\mathrm{echo}}}-\left[\mathrm{TR}-N_{\mathrm{echo}}\mathrm{TE}\right]\right)}_{N_{\mathrm{ph}}}$$

The subscript ‘x’ and ‘y’ with the $90^{{}^{\circ}}$-excitation and $180^{{}^{\circ}}$-refocusing pulses indicate a 90-degree phase difference. The term between curly brackets {} is the train of $180^{{}^{\circ}}$-refocusing pulses, resulting in $N_{\mathrm{echo}}$ echoes. A typical number of echoes in the vendor-provided multi-contrast pulse sequences is 32. The entire sequence is repeated for all $N_{\mathrm{ph}}$ phase encoding lines. The optimal number of echoes for maximum dose resolution is dependent on the dose-R_2_ characteristic of the polymer gel [74,76].

Essential to the accuracy of the dose distribution is the compensation for all possible sources of MR distortions in terms of both the acquired R_2_ value and the geometrical distortions [340]. A summary of MRI artifacts that can affect the uncertainty in polymer gel dosimetry is provided in Table 5.

Eddy-current-induced magnetic fields as a result of gradient switching may lead to both spatial distortions and inaccuracies in the estimated dose. Where eddy-current-induced magnetic field offsets during slice selection result in a slice shift in the slice selection direction, eddy current magnetic field offsets during readout result in a slice shift in the readout direction. The effect of eddy currents produced by imaging gradients in a multi-spin echo sequence may result in a misregistration between the different base images, which can result in erroneous R_2_ maps. The combination of eddy currents and stimulated echoes also leads to changes in slice profiles over the different base images, which eventually result in R_2_ deviations [64]. Stimulated echoes are the result of non-ideal slice profiles leading to a multitude of coherence pathways. It was shown experimentally and through Bloch simulations that the detrimental effect of eddy currents can be significantly reduced by the application of a gradient train before the excitation pulse [64].

B_0_ magnetic field inhomogeneities, gradient non-linearities and magnetic susceptibility differences can result in spatial distortions. B_0_ magnetic field inhomogeneities and gradient non-linearities can be measured by use of a grid phantom. Magnetic susceptibility differences between two media causes magnetic field distortions that depend on the shape of the phantom and its orientation in the external magnetic field. Magnetic susceptibility related geometric distortions can be compensated for by use of a magnetic field map that can be acquired by use of a susceptibility weighted echo time encoding (SWEET) sequence [67].

Although the fitted R_2_ is independent of the absolute value of the signal and, therefore, expected to be insensitive to B_1_-field non-uniformity, the interplay of stimulated echoes and B_1_-field inhomogeneity can still result in uncertainties. The effect of B_1_-field inhomogeneities can be minimized by using the body coil as transmitter coil. Remaining B_1_-field related inhomogeneities in the R_2_ map can be compensated for by using correction maps based on a measured B_1_-field map [65] or by applying a look-up table [69]. The correlation between B_1_-field imperfection and R_2_ can be derived experimentally using a blank (non-irradiated) gel phantom or, theoretically, from simulations based on the Bloch equations [65]. At magnetic field strengths in the order of 3T and above, depending on the shape and electrical properties of the gel phantom, standing waves may occur inside the phantom, which can lead to large non-uniformities in the B_1_-field. The occurrence of standing waves can be minimized by changing the dielectric properties of the gel dosimeter by adding salt to the gel.

As the R_2_ depends on the temperature during scanning, any variation in temperature during scanning results in an uncertainty. Temperature variations during scanning may be caused by temperature fluctuations in the scanner room or by scanning-induced RF heating. To minimize temperature related uncertainties, the gel dosimeter phantom should be placed in the scanner room several hours before scanning to equilibrate at the scanner room temperature [24,63]. To minimize the effect of RF-induced cooling, a centric k-space ordering scheme can be applied in the pulse sequence [66]. Temperature variations are the main contributor to uncertainties when polymer gel dosimetry is performed in an ‘absolute’ way [77].

#### 5.1.3. MT Mapping

The presence of microscopic magnetic field gradients induced by magnetic susceptibility differences in low-density lung-equivalent polymer gel systems results in a dispersion of the acquired R_2_ values resulting in very fast transverse decay. The very fast transverse decay (large R_2_) compromises the obtainable dose precision in gel foams. In this case, magnetization transfer can be exploited as a contrast mechanism that correlates with the absorbed radiation dose [328,341]. With magnetization transfer imaging, short echo times can be used, as the contrast is created by saturation of the macromolecular hydrogen pool by use of a train of RF pulses that are played out before the excitation pulse. On the other hand, the R_2_ dispersion can be simulated using a random walk model and has potential in the determination of microstructural parameters of the gel foam [342].

A more selective low-power off-resonance excitation in methacrylic acid-based polymer gels reveals a nuclear Overhauser effect (NOE) with hydrogen protons on the methyl group. The use of a NOE correlated dose map was illustrated for a brachytherapy irradiation source [62].

#### 5.1.4. Other MRI Techniques

As monomers become bound upon radiation, the peaks of the monomers in the NMR spectrum decrease [30]. By using a spin echo imaging pulse sequence with a band selective refocusing pulse centered on the resonances of the monomers, a spectral image of the monomers can be obtained. The low proton density of monomers inherently results in a low SNR, which needs to be compensated for by increasing the number of imaging averages [343].

Another physical property that changes in polymer gel dosimeters is the mechanical elasticity. The shear stiffness can be mapped by use of MR elastography (MRE). It is found that the shear stiffness in a methacrylic acid-based polymer gel dosimeter increases proportionally with the radiation dose, and a shear stiffness map of a square field was shown as a proof-of-principle [344,345]. While the precision in this preliminary study is poorer than with R_2_ imaging, MRE can play an important role in gel dosimetry with deformation.

### 5.2. Optical CT Scanning

**Figure 10:**
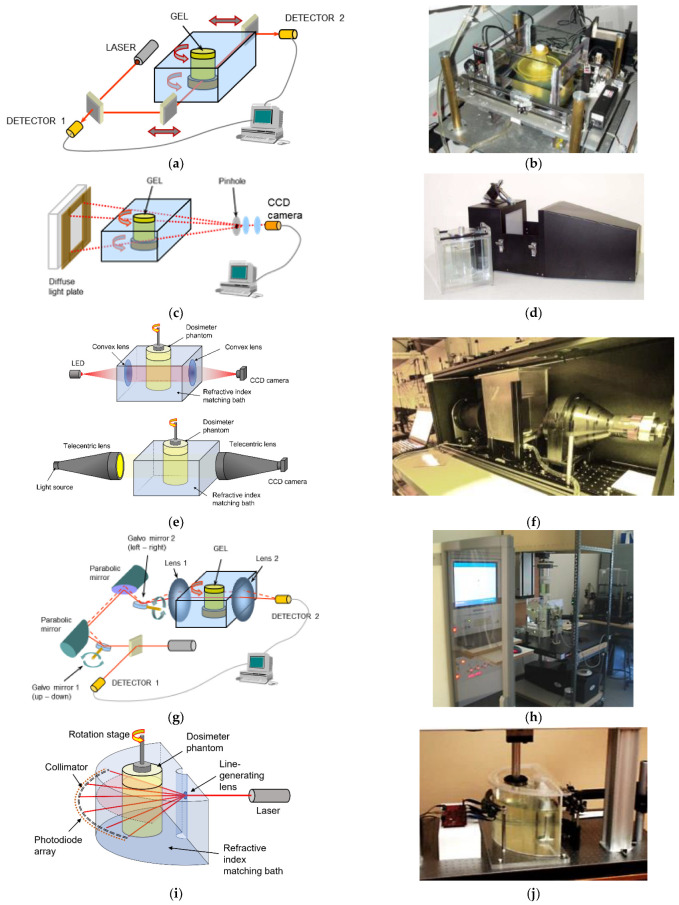

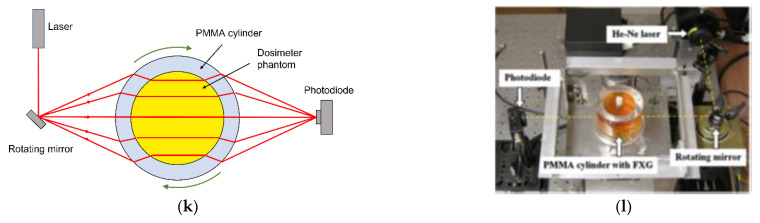
Various types of optical CT scanners: First generation laser scanner (**a**,**b**), cone beam optical CT scanner (**c**,**d**), second generation cone beam scanner with telecentric lenses (**e**,**f**), second generation optical laser scanner with galvanometer mirror and lens system (**g**,**h**), fan beam optical laser scanner (**i**,**j**) and dry laser scanner (**k**,**l**).

Different optical scanners have been developed in the last three decades. In early scanners, a scanning laser beam was applied. The advantage of such scanners lies in the fact that straight projections are acquired from different angles through the gel, filling up a sinogram matrix. Optical CT images can be easily reconstructed from the sinograms by use of a filtered back projection or a radon transform. First order light scattering can be compensated for by use of pinhole applicators. A disadvantage of laser beam scanners is that they contain moving parts that can introduce positional inaccuracies. Cone beam scanners where a flat light source is applied are currently the most preferred scanner type because of their high acquisition speed. Image reconstruction is performed by use of a cone beam back projection algorithm, which can be parallelized on computers containing a graphical processing unit (GPU) that is available for computation [346]. Specific aspects of optical CT scanning were reviewed elsewhere [84]. Different types of optical CT scanners were constructed and used to read out gel and radiochromic dosimeters (Figure 10).

The first generation of optical laser CT scanners consisted of a red laser and a set of moving mirrors that create a traveling laser beam through the dosimeter [81]. The phantom is mounted on a rotating turntable that is positioned in a square glass reservoir. To avoid deflection of the laser beam on the phantom, the glass reservoir is filled with a refractive index matching fluid (Figure 10a). Similar designs where the phantom is suspended from above were also designed and used [82,83,347]. For every incremental rotation, the laser beam is swept, and a light intensity profile is recorded. By using a half-reflecting mirror, the incident beam can be measured so that any fluctuations in the incident laser beam intensity are compensated for. After a full rotation of the phantom, the laser and mirror system are moved upward, and a new slice is recorded. The procedure is repeated until the entire volume is scanned. A disadvantage of this type of scanner is the relatively long imaging time and the susceptibility of the scanning to mechanical vibrations from the moving parts. Laser scanners were commercialized by the company MGS Research Inc. (Madison, CT, USA) under the tradename “Octopus™”. Faster scanning is achieved by use of a cone beam optical CT scanner (Figure 10b) where a diffuse light source is used and a CCD camera collects an entire image for each rotation. The entire phantom is scanned after one rotation of the phantom [348]. To eliminate primary light scatter, a pinhole is applied before the CCD camera. This kind of scanner was commercialized by Modus QA (London, ON, Canada) under the trade name “Vista™”. The time to scan an entire phantom is reduced to a few minutes with cone beam optical scanners, whereas laser scanner systems would typically take a few hours. Some modifications to the cone beam optical scanner were implemented to allow scanning at different wavelengths and with higher bit depth [194]. To minimize image artifacts from secondary light scatter, a parallel beam optical scanner was introduced, in which a big lens is employed to create a parallel beam of light incident from a mercury lamp. The parallel beam projects a transmission image on a diffuser screen, which is captured by a CCD camera [85] (Figure 10e,f). This design was further improved by employing two telecentric lenses [349,350]. With this design, only rays of light that are orthogonal to the second telecentric lens are captured, filtering out any scattered light from other directions. The first generation of optical laser scanning was improved by replacing the translating mirrors and vertical stage with Galvano mirrors and large plano-convex lenses that create a sweeping laser ray that travels through the fluid tank and dosimeter phantom (Figure 10g,h) [351]. To compensate for nonuniform optical aberration effects in the vertical direction and to increase the maximum phantom size, a modification was made by Vandecasteele and De Deene as the vertical deflecting Galvano mirror was replaced by a linear stage that moves the phantom vertically with respect to the laser scanning plane [86,169]. In a fan-beam optical CT scanner [87], a fan beam is obtained by use of a pinhole and lens, which is collected by a circular array of detectors after passing through a semi-cylindrical fluid tank in which the phantom is placed (Figure 10i,j). Attempts were made to remove the need for a refractive index matching fluid by using a cylindrical shell of PMMA that encloses the dosimeter phantom and by making use of the light-focusing effect of the cylindrical geometry [352,353], by adapting the reconstruction algorithm [354] or by use of aspherical lenses [355]. It can be concluded that with all ‘dry’ methods, the outer portion of the phantom cannot be scanned.

The main challenge with the optical CT scanning of polymer gel dosimeters is the diffuse light scattering by the irradiated dosimeter [346], as light scattered in a different direction from the primary beam can end up in the detector. Cone beam optical scanners are the most sensitive to light scattering [356,357] while double telecentric lens scanners and laser scanning systems are the least sensitive [349].

### 5.3. X-ray CT Scanning

A small radiation-induced change in electron density was detected in polymer gel dosimeters. This change is attributed to the expulsion of water from the precipitating polymer aggregates [57]. The dose-dependent change in CT number expressed in Hounsfield units (H) enables the use of X-ray CT scanning [78,79,80,287]. The relatively low dose sensitivity is one of the major challenges of X-ray CT scanning of polymer gel dosimeters, and several studies were conducted to find a polymer gel recipe that results in the highest dose sensitivity. The dose-CT number response follows a mono-exponential saturation response, which at low doses, can be approximated by a linear function.

The change in CT number for a (6%T/50%C) anoxic PAG gel amounts to 0.86 H Gy^−1^, but some inter-batch variation was reported [78,358]. It was also found that the dose sensitivity decreased with increasing gelatine concentration and increased with increasing monomer concentration [358]. The use of an antioxidant decreases the dose sensitivity. It is postulated that the reaction of THP with amine groups on the gelatine molecules is responsible for the reduced dose sensitivity [38]. For PAGAT gel (6%T/50%C), the dose sensitivity is 0.31 H Gy^−1^ [359] and for MAGAT gel (9% MAc; 8% gelatin), a value of 0.85 H Gy^−1^ is reported [359]. For MAGIC gel (9% Mac; 8% gelatin), the dose sensitivity is 0.38 H Gy^−1^ [360]. The use of cosolvents such as isopropanol and glycerol were explored with the aim to increase the amount of cross-linker that could be dissolved in the gel to increase the dose sensitivity [361]. Glycerol also increased the dose sensitivity on itself but had a negative impact on the linearity of the gel dosimeter [362]. In this study, the less toxic N-isopropylacrylamide (NIPAM) was used as an alternative for acrylamide. It was later found that NIPAM also increases the solubility of the cross-linker Bis, and a dose sensitivity of 0.88 H Gy^−1^ was determined for a 15% NIPAM/4.5% Bis gel dosimeter without cross-linker [363], which is similar to the highest sensitivity achieved with cosolvents. It was also found that a higher density change was obtained at lower and higher relative cross-linker concentrations (%C) than at intermediate %C [364], which is contrary to the trend in NMR dose sensitivity where the maximum dose sensitivity is found for 50%C polymer gel dosimeters [43,50]. However, because of the non-linearity of the dose response at low %C, a 50%C gel formulation may still be preferred for X-ray CT polymer gel dosimetry.

To compensate for the relatively low dose sensitivity in X-ray CT, many image averages are typically taken as the SNR is proportional to the square root of the number of averages [78]. The number of image averages is traded off against the thermal burden on the X-ray tube and the total imaging time. As the CT scanning itself also contributes some extra dose, a ‘zero-scan’ method is used, whereby the change in CT number response over the several base images is filtered out [365]. Beyond maximizing the dose sensitivity of the polymer gel and taking several image averages, other strategies to increase the SNR in X-ray CT scanning are taken. Scanning parameters can be optimized [366,367], and it is concluded that minimizing phantom size and maximizing tube voltage are recommended. In the light of scanning parameter optimization, a conventional X-ray computed tomography scanner was modeled by use of a Monte Carlo simulation [368]. An additional strategy to increase the SNR in the X-ray derived dose maps is the use of post-processing image filtering techniques [369,370,371].

Radiation properties, such as temporal stability, spatial integrity and dose-rate dependence, were also determined for NIPAM polymer gel dosimeters [372,373]. With a temperature dependence of 0.5 % (°C)^−1^, X-ray CT imaging is less sensitive to the temperature during imaging than MRI [78]. The effect of parameter optimization and fitting of calibration data on the accuracy in calibrated images was documented [374].

The applications of polymer gel dosimetry with an X-ray CT readout were demonstrated for conventional photon treatment beams [375], stereotactic radiosurgery [78], IMRT [372,376], proton beams [377] and radiation delivery during deformation [378]. Polymer gel dosimetry was also read out with the Linac-integrated kV-cone beam CT modality [379], making it an interesting tool for IGRT.

### 5.4. Other Scanning Methods

Any physical property that is affected by the radiation-induced reaction can be exploited as a potential non-destructive imaging technique. As it was found that the acoustic properties of polymer gel dosimeters, such as speed of sound propagation and attenuation, change upon irradiation [380,381], ultrasonic imaging was pursued [88]. The change in the speed of sound in irradiated polymer gel dosimeters is attributed to the change in elasticity modulus and mass density as a result of the formation of polymer aggregates [382,383]. Additional studies on the frequency dependence of the ultrasonic attenuation coefficient were conducted [384]. The feasibility of vibro-acoustic imaging, whereby a burst of focused ultrasound causes a local vibration at a different frequency, was also demonstrated [385]. Other possible imaging techniques, such as electrical impedance tomography (EIT), diffuse optical tomography (DOT) and photoacoustic imaging, have not been explored yet.

## 6. Uncertainty in 3D Radiation Dosimetry

The measurement uncertainty of a single dosimetry experiment comprises both systematic and random errors. Uncertainties can be classified in type A and type B uncertainties where type A standard uncertainty is obtained from a probability density function derived from an observed frequency distribution, while type B standard uncertainty is obtained from an assumed probability density function that is based on the degree of belief that an event will occur. A measure of the uncertainty in a dosimetry experiment can be achieved through a reproducibility study of the complete dosimetry experiment from gel fabrication to dose distribution analysis (type A uncertainties) and by comparison against other dosimetry standards (type B uncertainties) [77].

The uncertainty analysis in a 3D radiation dosimetry experiment is further complicated as the spatial and dosimetric dimensions are interwoven, and it is theoretically impossible to extract both dosimetric and spatial errors from a measured spatial dose. It is in this light that gamma-map comparisons were used [267,268].

A clinical 3D dose verification experiment is performed in different steps, from manufacturing the gel to irradiation and reading out the dosimeter, each adding to the overall uncertainty. Type A uncertainty is in relative terms (percentage of the maximum dose in the dose distribution) dependent on the dose sensitivity of the gel (optical absorbance, R_1_, R_2_, CT number, etc.) and the stochastic noise in the acquired images. The noise level is also intricately connected with the spatial resolution (voxel size) and measurement time (number of image averages). With an MRI readout, type A uncertainties can be minimized by optimizing the imaging parameters, such as repetition time, echo time and flip angle [74,75,76]. With optical CT, the light source intensity, integration time of the camera or detector and step size can be optimized, while in X-ray CT, the tube current and tube potential can be optimized, but here also, the thermal load of the tube needs to be considered in this regard. The optimal imaging parameters are dependent on the type of gel (i.e., the R_1_ and R_2_ working range of the gel dosimeter, the range of optical absorbance and X-ray attenuation). Type B uncertainties can have a physico-chemical origin (such as temperature dependence, dose-rate dependence and instability) or are attributed to imaging artifacts. To minimize type B uncertainties of physico-chemical origin, gel dosimeters with favorable physico-chemical properties should be used and temperature and fabrication procedures should be controlled carefully. MRI artifacts can be minimized by a careful choice of pulse sequences and artifact compensation techniques [64,65,66]. With every dosimeter and imaging modality it is crucial to follow a carefully optimized protocol and compensate for imaging artifacts to minimize the uncertainties [63,64,65,66,67].

The intricate coupling of the imaging accuracy and precision with the physico-chemical properties of the 3D dosimeters complicates a quantitative comparison. Moreover, the contribution of the different physico-chemical properties to the overall uncertainty figure depends on the dose range and the dose-rate distribution in the treatment. For a more extensive discussion of the uncertainty in 3D radiation dosimetry the reader is referred to other publications [23,24,25,63,77].

## 7. Towards 4D Radiation Dosimetry

To increase the tumor-conformity, with the reduction in treatment margins that accommodate for set-up uncertainties, several image guided radiotherapy (IGRT) techniques have been introduced since the end of the 20th century [386]. Three different levels of incorporation of IGRT in clinical practice are considered by the International Atomic Energy Agency (IAEA) [387]: Level 1 involves the off-line review of megavoltage (MV) electronic portal images (EPIDs) or radiographic film. Level 2 involves paired kilovoltage (kV) imaging and automated analysis of patient and organ shifts in order to assess action levels at the start of each treatment fraction. Interfraction changes may result from tumor regression or growth, changes in patient weight and variations in patient position. Level 3 incorporates motion management and involves intrafraction target visualization. Intrafraction changes may be the result of respiration and cardiac motion, peristalsis and short-period movements such as coughing, muscular spasms and wriggling. Various imaging techniques can be used in IGRT, such as topographic X-ray, X-ray CT, MRI and ultrasound. Earlier techniques of IGRT used kV and MV X-rays and had the disadvantage that they delivered some additional radiation to the patient. While this dose from imaging is relatively small compared to the dose from treatment, the radiation from imaging is delivered over a larger area, which is especially of concern in pediatric patients and limits the use for interfractional adaptation. With the introduction of the MR-Linac, it is now possible to acquire images with superior soft-tissue contrast before the start of each treatment fraction without delivering any ionizing radiation to the patient. This class of treatments is referred to as ‘MR-guided radiotherapy’ (MRgRT). Many groups have started with the development and implementation of fast MRI sequences with sub-second temporal resolution that would enable intrafraction adaptation [388,389]. Beyond anatomical imaging, MRI can also provide bio-functional images that can be used to adapt the dose prescription during fractionated treatment on the basis of biological tumor response. The prescription of dose on the basis of quantitative biomarkers is also referred to as ‘dose painting’.

An important element in any IGRT program is the development and implementation of dosimetric QA. The need for end-to-end 3D dosimetry is emphasized with the introduction of IGRT in the treatment chain. In this respect, gel dosimetry is likely to play a unique role as it is the only dosimeter that is able to acquire the dose distribution in full 3D with realistic human anatomy.

Preliminary studies demonstrated the feasibility of the use of 3D gel dosimetry for the observation of the effect of a gated treatment on the dose distribution [274,390,391,392]. In these studies, gating was achieved by use of a commercial tracking system (Varian Real-time Position Management) employing external optical imaging of external markers [274,388,389] or by use of pressure sensors [392] from which the motion was inferred. Motion was induced by use of an in-house constructed moving platform [390], a linear stage [274], a commercial dynamic phantom (CIRS, Sun Nuclear) [391] and inflation of a porcine lung [383]. Both polymer gel dosimeters [274,390,392] and 3D radiochromic dosimeters [391] were used in these studies.

With cone beam IGRT and MRgRT, it is essential that a common isocenter and coordinate system for both imaging and treatment is applied. MRI is also prone to geometrical image distortions as a result of magnetic field heterogeneity and gradient non-linearity. Magnetic field distortions can also arise from magnetic susceptibility differences between air and tissue. Gel dosimetry can be employed to assess and correct the alignment of the isocenter and to assess image distortions [393,394].

The strong magnetic field of the MRI subunit on MR-Linacs has an influence on the performance of many dosimeters. Studies were conducted to investigate the possible influence of the magnetic field on the dose response of 3D dosimeters and to gain confidence on the useability of an MR-Linac system on polymer gel dosimeters [93,395,396,397,398], radiochromic dosimeters [197,199] and Fricke gel dosimeters [399]. No detectable effect of the magnetic field on the dose-response was found in any of the 3D dosimeters. As discussed in the previous sections, in chemical 3D dosimeters, other effects may induce uncertainties in the measured dose distribution if not compensated for. Therefore, it is important to optimize the dosimetry protocol for each of the gel dosimeter systems.

The feasibility of MR-guided compensation of interfractional displacement was demonstrated in a cylindrical phantom containing polymer-gel-filled and tissue-equivalent anthropomorphic shaped structures [400]. The anthropomorphic shaped recipients were constructed by use of additive manufacturing techniques [401]. End-to-end testing of MRgRT was demonstrated on a deformable anthropomorphic pelvis phantom containing a rigid polymer gel dosimeter, which was irradiated in five treatment fractions with different filling of the bladder and rectum [402].

While previous studies demonstrated the ability of dosimetry in situations of organ motion, the tissue in patients may also be deformed. An interesting feature of gel systems is their capability for elastic deformation. The effect of deformation on the dose distribution was studied in polymer gel dosimeters [403,404], a flexible radiochromic silicone dosimeter [200] and a deformable PRESAGE™ polyurethane dosimeter [405]. These deformable 3D dosimeters can play a crucial role in the assessment of computational deformable image registration (DIR) algorithms [406].

**Figure 11:**
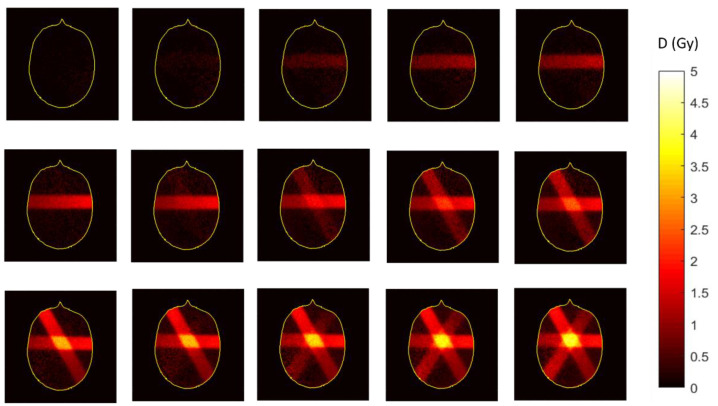
Sequential dose maps of a central slice in a MAGAT gel dosimeter recorded during treatment delivery by use of the MRI subunit on an MR-Linac [93]. The time between two adjacent images in a row is 44 s (every 4th recorded frame). Adapted from figure [93] with permission from Institute of Physics and Engineering in Medicine, Copyright 2000 IOP Publishing.

Attempts have been undertaken to acquire dose maps with Fricke and polymer gel dosimeters in real-time during treatment delivery on an MR-Linac using the MRI subunit for dose readout [93,163,398] (Figure 11).

As with any imaging technique, there is a trade-off between temporal resolution and signal-to-noise ratio (SNR), which can be comprised in the concept of temporal uncertainty. For real-time imaging with gel dosimeters (Figure 11), it is paramount that the dose sensitivity is sufficiently high so that the change in dose between two image frames is detectable. The temporal uncertainty (with confidence level 95%) for the three different gel dosimeters employed in previous studies (compared at similar dose rates) are 106 s for a ferrous oxide xylenol orange gel [163], 27 s for the VIPET gel dosimeter [398] and 3.8 s for the MAGAT gel dosimeter [93]. While these first results do not provide dose maps at sub-second resolution, there is room for improvement by a further increase in the dose sensitivity and in the quantitative MRI imaging approach by exploiting the sparsity of information in dose distributions. It is also important to remain vigilant on the radiation properties of the gel dosimeters. Polymer gel dosimeters demonstrate post-irradiation polymerization, which results in a retardation in the dose registration, and the dose-rate dependence of MAGAT gel dosimeters is traded in for a high sensitivity [93]. From these studies, it can be concluded that new gel formulations with high-dose sensitivity and favorable radiological and dosimetric characteristics are needed to register the dose at sub-second time intervals [407].

## 8. Conclusions and Outlook to the Future

Gel dosimetry is an active field of research that was developed in light of a need for 3D dose registration in emerging radiotherapy techniques that aim to deliver higher levels of radiation to solid tumors while minimizing the dose to surrounding healthy tissue, in particular to radiation sensitive critical organs. Many different radiation-sensitive gel systems were fabricated and evaluated. As a dosimetric measurement technique for the quality assurance of cancer therapy, the gel dosimeters need to satisfy several radiophysical properties, such as tissue equivalence, dose-rate independence, temperature independence, energy independence, stability and spatial integrity. In addition to the development of gel dosimeters, different imaging methods were developed and optimized to read out the gel dosimeters with high precision and accuracy.

Some gel dosimeters were applied successfully in a variety of radiotherapy techniques, including but not limited to IMRT, IMAT, Tomotherapy^®^ and brachytherapy. Despite a demonstrated record of some important clinical applications, gel dosimetry has not entirely found its way from lab to bedside, apart from the successful implementation in a handful of radiotherapy centers. Contributing factors to the poor dissemination in clinical practice is the perception in the medical physics community that gel dosimetry is relatively labor intensive, time-consuming and requires a significant amount of scientific expertise with respect to the chemistry of gel fabrication and the imaging techniques to read out the gel dosimeters. The large variety of proposed gel dosimetry systems and different readout methods is also perceived as daunting to medical radiation physicists who are looking for a turn-key solution. Additionally, over the years, gel dosimetry has seen competition from the introduction of commercial electronic dosimetric QA systems that are able to measure the absorbed dose in multiple discrete points in space. While these electronic systems are not fully three-dimensional and do not provide an end-to-end test of the entire treatment chain in anthropomorphic geometries, their ease-of-use has made them more attractive in the medical physics community.

While the poor dissemination of 3D gel dosimetry in the clinic is multi-factorial, the author is of the opinion that the gel dosimetry research community could help medical radiation physicists with the implementation of 3D gel dosimetry. Paramount in the implementation of gel dosimetry in the clinic is an assessment of the uncertainty of both the 3D gel dosimeter and the readout system on site followed by a dose-verification in three dimensions whereby the readout method is optimized for maximum dose resolution and minimal MRI artifacts.

With the current introduction of image guided radiotherapy (IGRT) techniques and theranostic MR-Linac systems, it is likely that interest in gel dosimetry will resurface because of the unique features of gel dosimeters as end-to-end dosimetric QA, deformable dose registration and anthropomorphic geometry. The ability of reading out the dosimeter in real time with the use of the MRI subunit on MR-Linacs is another great advantage.

## Figures and Tables

**Table 1:** The radiolytic yield of primary water products at neutral pH and for a 0.8 N H_2_SO_4_ aqueous solution [106]. G values are also given in SI units.

Radiolytic Products	Neutral pH		0.8 N H_2_SO_4_ Aqueous Solution	
	G (Part./100 eV)	G (µmol.J^−1^)	G (Part./100 eV)	G (µmol.J^−1^)
$e_{\mathrm{aq}}^{-}$	2.65	0.274	3.7 ^a^	0.38 ^a^
H^•^	0.6	0.062		
$H_{2}$	0.45	0.047	0.4	0.041
$H_{2}O_{2}$	0.68	0.070	0.8	0.083
OH^•^	2.8	0.29	2.9	0.30
$H_{2}O^{-}$	4.15	0.43	4.5	0.47

^a^ The first row of values specified for a 0.8 N sulfuric acid solution are for the sum of the aquatic electron ($e_{\mathrm{aq}}^{-}$) and the hydrogen radical (H^•^). In a sulfuric acid solution with concentration above 0.4 N, the aquatic electron rapidly reacts with hydroxonium, resulting in a conversion of 99.9% of the hydrogen radical after 1 µs.

**Table 2:** Studies reporting chemical factors, radiation properties and NMR properties that affect the dose response of Fricke NMR gel dosimeters.

Factor	Agarose	Gelatine
***Chemical properties***		
Gelling agent	[10,112,116]	[111,113,118,119]
pH, [H_2_SO_4_]	[110,112]	[113,118,119]
Initial Fe^2+^ concentration	[110,112]	[120]
NaCl	[117]	[121]
Other additives (saccharides)	[10,115,117]	[113]
O_2_	[110,111,112]	[113,121]
Cooling rate	[110]	
***Radiation properties***		
Post-irradiation time	[110]	[113,121]
Dose rate	[111,112]	
Beam energy	[110,122]	[122]
Tissue equivalence	[110,122]	[122]
***NMR properties***		
NMR frequency	[112]	[113]
Multi-exponential relaxation	[123]	

**Table 3:** Different monomers used in polymer gel dosimeters and the corresponding half-value dose, R_2_-dose sensitivity and dynamic R_2_-range. AAm, VP, HEA, HEMA and NIPAM are all used in combination with the cross-linker Bis (shaded). AAc and Mac are used without the cross-linker. (N/A = Not applicable).

Monomer	Chemical Formula	D_1/2_ (Gy)	R_2_-Dose Sensitivity (s^−1^.Gy^−1^)	R_2sat_-R_20_ (s^−1^.Gy^−1^)	Ref.
Acrylamide (AAm)	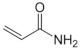	5.5 (±0.1)	0.331 (±0.012)	4.2 (±0.4)	[45]
1-Vinyl-2-Pyrrolidone (VP)	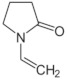	23.6 (±0.1)	0.082 (±0.004)	13.7 (±0.4)	[31,234]
2-Hydroxyethyl Acrylate (HEA)	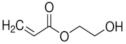	5.5 (±0.1)	0.498 (±0.003)	4.2 (±0.4)	[31,45,235]
2-Hydroxyethyl Methacrylate (HEMA)	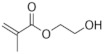	41.6 (±0.1)	0.046 (±0.002)	4.9 (±0.4)	[31]
N-iso-propyl-acrylamide (NIPAM)	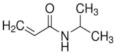	10	0.13 (±0.012)	4.2 (±0.4)	[236]
N,N’-methylene-Bis-acrylamide (Bis)	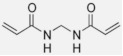	N/A	N/A	N/A	N/A
Acrylic Acid (AAc)	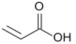	31.2 (±0.1)	0.358 (±0.006)	10.6 (±0.4)	[31]
Methacrylic Acid (MAc)	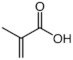	12.5 (±0.1)	1.193 (±0.048)	18.4 (±0.4)	[31,43,45]

**Table 4:** Cross reference table to studies on radiation properties of different classes of polymer gel dosimeters.

Gel Type	Stability	Spatial Integrity	Dose Rate	Energy	Temp. Irradiation	Temp. Scanning	Temp. Fabric./Shelf Life	Tissue Equiv.
PAG	[28,43,45,51]	[43,45,47]	[43]	[43,240]	[43]	[43,50,61,241]		[43,242]
AAG			[243]	[243]				
PAGAT	[43,55]	[43,55]	[43]	[43]	[23,43]	[43]	[23,44]	[43,45]
MAGAT	[43]	[43]	[43]	[43]	[43]	[43]	[44]	[43,240]
MAGIC	[45]	[45]	[49]					[240]
ABAGIC	[45]	[45]						
NIPAM	[244]			[245]				[245]
VIPAR	[246]	[246]	[246]				[246]	
VIPARnd	[247]	[247]	[247]	[247]				
NIBMAGAT			[248]				[248]	
NHMAGAT	[249]			[249,250]		[249,250]	[250]	
MAGADIT			[251]					
PAMPSGAT	[252,253]		[254]	[254]		[252,253]		[252,253,254]
NMPAGAT			[255,256]	[255]	[256]	[256]	[256]	

**Composition Polymer Gel Type (acronyms).**
PAG: Acrylamide (AAm)/N,N’-methylene-Bis-Acrylamide (Bis)/Gelatine/Nitrogen purged. AAG: Acrylic Acid (AAc)/Bis/Gelatine/NaOH. PAGAT: AAm/Bis/Gelatine/Tetrakis(hydroxymethyl)phosphonium salt (THP). MAGAT: Methacrylic acid (MAc)/Gelatine/THP. MAGIC: MAc/Gelatine/Ascorbic acid (AscA)/Copper sulphate/(hydroquinone (HQ)). ABAGIC: AAm/Bis/Gelatine/AscA/Copper sulphate. NIPAM: N-isopropylacrylamide/Bis/Gelatine/(THP). VIPAR: N-vinylpyrrolidine (NVP)/Bis/Gelatine/Nitrogen or Argon purged/(isopropanol). VIPARnd: NVP/Bis/Gelatine/AscA/Copper sulphate/(isopropanol)/(tert-butanol)/(HQ). NIBMAGAT: N-isobutoxymethylacrylamide (NIBMA)/Bis/Gelatine/THP/(glycerol, acetone, methanol). NHMAGAT: N-(hydroxymethyl)acrylamide (NHMA)/Bis/Gelatine/THP/(CaCl_2_). MAGADIT: MAc/Gelatine/Dithiothreitol (oxygen scavenger). PAMPSGAT: 2-Acrylamido 2-Methyl Propane Sulfonic acid (AMPS) or salt/Bis/Gelatine/THP/NaOH. NMPAGAT: N-(3-Methoxypropyl)acrylamide (NMPA)/Bis/Gelatine/Glycerol/THP.

**Table 5:** Overview of important MRI artifacts that may compromise the accuracy of quantitative R_2_ maps and the derived dose maps [340]. The artifact sources are classified based on their effect (geometrical distortions versus dose inaccuracies) and origin (machine related or object related).

**Geometrical Distortions**		**Dose Inaccuracies**	
**Machine Related**	**Object Related**	**Machine Related**	**Object Related**
Magnetic field Heterogeneity	Magnetic susceptibility differences	Eddy currents	Temperature drift
Magnetic gradient non-uniformity	Chemical shifts	Stimulated echoes	Molecular self-diffusion
Eddy currents		RF-field inhomogeneity	
		Imperfect slice profile	
		Standing waves	

## Data Availability

Not applicable.
